# Ubiquitin ligase RNF20/40 facilitates spindle assembly and promotes breast carcinogenesis through stabilizing motor protein Eg5

**DOI:** 10.1038/ncomms12648

**Published:** 2016-08-25

**Authors:** Yang Duan, Dawei Huo, Jie Gao, Heng Wu, Zheng Ye, Zhe Liu, Kai Zhang, Lin Shan, Xing Zhou, Yue Wang, Dongxue Su, Xiang Ding, Lei Shi, Yan Wang, Yongfeng Shang, Chenghao Xuan

**Affiliations:** 1 Department of Biochemistry and Molecular Biology, Tianjin Key Laboratory of Medical Epigenetics, Tianjin Medical University, Tianjin 300070, China; 2 Tianjin Key Laboratory of Lung Cancer Metastasis and Tumour Microenvironment, Tianjin Lung Cancer Institute, Tianjin Medical University General Hospital, Tianjin 300052, China; 3 Department of Immunology, Tianjin Medical University, Tianjin 300070, China; 4 Institute of Biophysics, Chinese Academy of Sciences, Beijing 100101, China; 5 Department of Biochemistry and Molecular Biology, Key Laboratory of Carcinogenesis and Translational Research (Ministry of Education), Peking University Health Science Center, Beijing 100191, China

## Abstract

Whether transcriptional regulators are functionally involved in mitosis is a fundamental question in cell biology. Here we report that the RNF20/40 complex, a major ubiquitin ligase catalysing histone H2B monoubiquitination, interacts with the motor protein Eg5 during mitosis and participates in spindle assembly. We show that the RNF20/40 complex monoubiquitinates and stabilizes Eg5. Loss of RNF20/40 results in spindle assembly defects, cell cycle arrest and apoptosis. Consistently, depletion of either RNF20/40 or Eg5 suppresses breast cancer *in vivo*. Significantly, RNF20/40 and Eg5 are concurrently upregulated in human breast carcinomas and high Eg5 expression is associated with poorer overall survival of patients with luminal A, or B, breast cancer. Our study uncovers an important spindle assembly role of the RNF20/40 complex, and implicates the RNF20/40-Eg5 axis in breast carcinogenesis, supporting the pursuit of these proteins as potential targets for breast cancer therapeutic interventions.

## Abstract (web summary)

Eg5 has a role in spindle assembly and has been associated with tumorigenesis but it is not clear how its activity is regulated. Here, the authors show that the E3 ligase RNF20/40 regulates mitotic spindle assembly by regulating the stability of Eg5 through mono-ubiquitination of K745.

## Graphical abstract

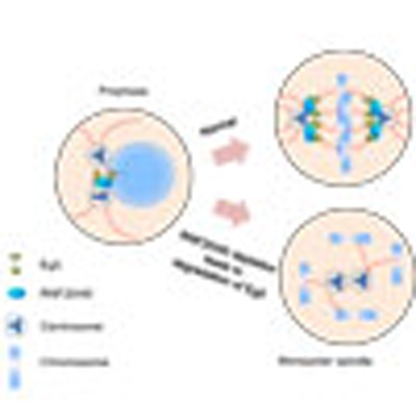

Mitosis is a highly orchestrated process involving numerous factors. At the onset of mitosis, accompanied by nuclear envelope breakdown, chromosomes condense and sister chromatids are segregated^1^. The condensation of interphase chromatin into mitotic chromosomes restricts transcription factor and chromatin remodelling factor access to DNA, rendering cessation of transcription^2^,^3^. Accurate chromosome segregation is essential for genome stability and cellular fitness. Chromosome missegregation may lead to lethality, aneuploidy or malignant cell transformation^4^,^5^. The mitotic spindle, a bipolar apparatus composed of microtubules and associated proteins thereof, is crucial to accurate chromosome segregation which relies on microtubule-dependent motor proteins^6^. Eg5, a plus-end-directed microtubule motor protein that is localized along spindle microtubule and near spindle poles, is enriched, plays important roles in bipolar spindle assembly and centrosome separation^7^. Although Eg5 spindle localization is consistent with its mitotic function, Eg5 concentration at spindle poles coincides with the possibility that Eg5 functions on both parallel and antiparallel microtubules^7^, which is supported by the observations that Eg5 inhibition leads to disruption of centrosomes and spindle poles^8^. As a kinesin family member, Eg5 has a highly conserved N-terminal catalytic motor domain and a C-terminal microtubule-binding domain^9^. Eg5 activity inhibition hinders centrosome separation during prophase and blocks bipolar spindle assembly during prometaphase^10^,^11^. Consequently, Eg5 inhibition leads to cell cycle arrest in mitosis with a monopolar spindle^12^,^13^,^14^, resulting in cell apoptosis^15^,^16^,^17^. Interestingly, Eg5 expression is upregulated in various malignancies including lung, bladder and pancreatic cancers^18^,^19^,^20^,^21^. Due to its pivotal bipolar spindle assembly and tumorigenesis roles, Eg5 has attracted much attention as a therapeutic target for cancers^22^. However, how Eg5 expression/activity is regulated is poorly understood, and its role in breast carcinogenesis has not been explored.

The RNF20/40 complex is composed of two Bre1 homologues, RNF20 and RNF40, both containing a C-terminal RING-finger domain. These two proteins form a tight heterodimer that is the major E3 ligase responsible for histone H2B lysine (K) 120 monoubiquitination (H2BK120ub) in mammalian cells^23^,^24^. H2BK120 monoubiquitination facilitates the methylation of histone H3 at K4 and K79 that are associated with active transcription, accounting for the role of the RNF20/40 complex in transcriptional regulation^25^,^26^,^27^. Interestingly, the RNF20/40 complex has also been implicated in tumorigenesis. RNF20 or RNF40 knockdown inhibits the growth of different types of mouse cells^28^ as well as human LNCaP cells^29^, and RNF20 depletion suppresses leukaemia progression *in vivo*^30^. Whether, and how, the RNF20/40 complex is functionally involved in mitosis, when it is supposed to be excluded from chromatin, is not clear; whether, and how, RNF20/40 E3 ligase plays a role in breast carcinogenesis is not understood.

In the current study, we find that the RNF20/40 complex participates in mitotic spindle assembly by interacting with, monoubiquitinating, and therefore stabilizing Eg5. We show that RNF20/40 loss-of-function leads to Eg5 destabilization, spindle assembly defects, cell cycle arrest and apoptosis. We demonstrate that either RNF20/40 or Eg5 depletion in breast cancer cells inhibits breast cancer growth in athymic mice. We also find that RNF20/40 and Eg5 are concurrently upregulated in human breast cancer samples and that high Eg5 expression is associated with worse overall survival of patients with luminal A, or B, breast cancer.

## Results

### Ubiquitin ligase RNF20/40 complex interacts with Eg5

**Figure 1:**
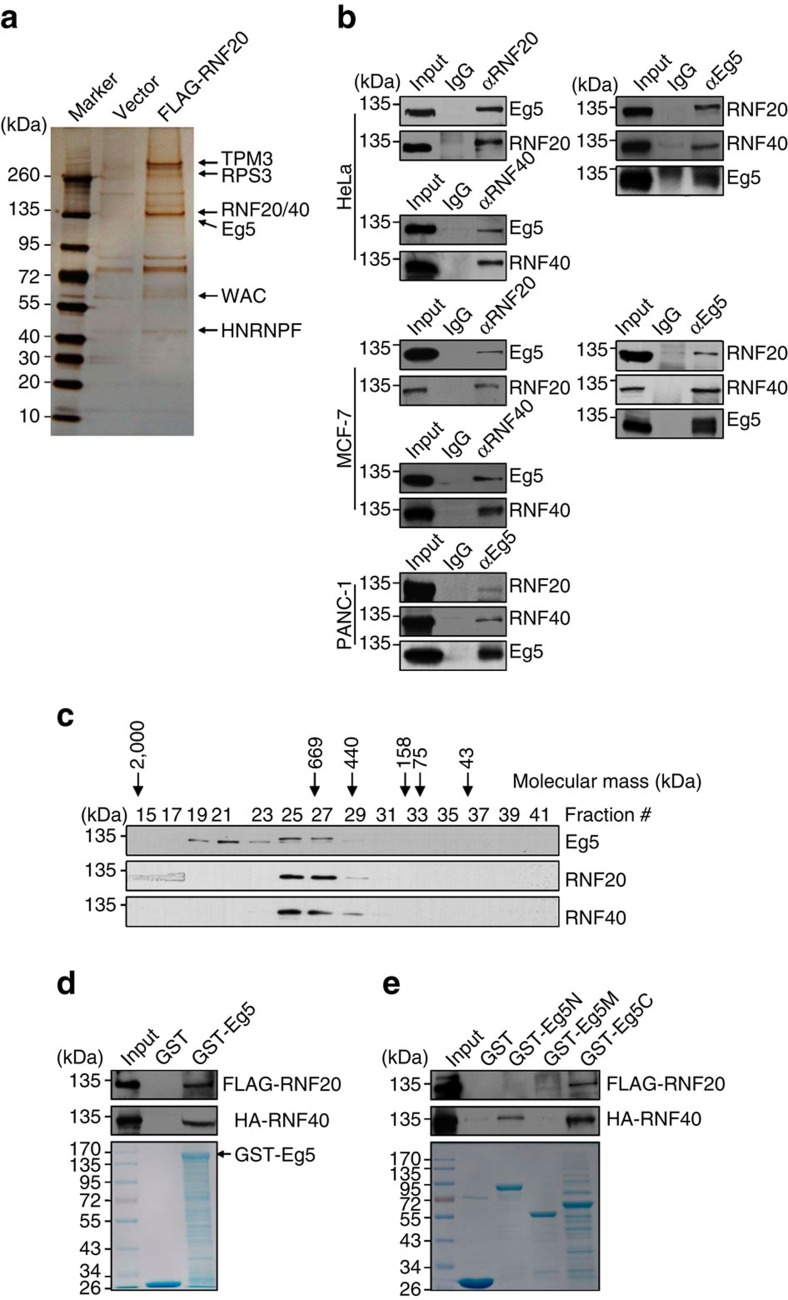
Ubiquitin ligase RNF20/40 interacts with the motor protein Eg5. (**a**) Immunoaffinity purification of RNF20-containing protein complexes. Cellular extracts from HeLa cells stably expressing FLAG (vector) or FLAG-RNF20 were immunopurified with anti-FLAG affinity columns and eluted with FLAG peptides. The eluates were resolved by SDS–PAGE and silver-stained. The protein bands were retrieved and analysed by mass spectrometry. (**b**) The RNF20/40 complex interacts with Eg5 *in vivo*. Immunoprecipitation assays were performed with antibodies against the indicated proteins followed by immunoblotting in HeLa, MCF-7, and PANC-1 cells. (**c**) Co-fractionation of Eg5 and the RNF20/40 complex by fast protein liquid chromatography. Cellular extracts from MCF-7 cells were fractionated on Superose 6 size exclusion column. The chromatographic profile with the elution positions of calibrating proteins of known molecular mass is shown. The chromatographic fractions were analysed by western blotting with antibodies against indicated proteins. (**d**) Eg5 interacts directly with RNF20 and RNF40 *in vitro*. GST pull-down assays were performed with GST-Eg5 and *in vitro* transcribed/translated RNF20 and RNF40. (**e**) Identification of the domains responsible for the direct interaction between Eg5 and RNF20 or RNF40. Bacterially purified GST, GST-Eg5N, GST-Eg5M or GST-Eg5C was incubated with *in vitro* transcribed/translated RNF20 or RNF40, after which GST pull-down assays were performed.

Functional analyses of the RNF20/40 complex have so far focused on its role in catalysing the monoubiquitination of histone H2B^23^,^24^. In an effort to further explore the biological activity of this complex, affinity purification and mass spectrometry were applied to identify proteins that are potentially associated with RNF20/40 *in vivo*. For this purpose, the lysates of HeLa cells expressing FLAG-RNF20 were prepared and subjected to FLAG affinity purification. The eluates were resolved on SDS–PAGE and silver-stained (Fig. 1a). Mass spectrometric analysis of the resolved protein bands indicated that RNF20 co-purified with RNF40, as expected, and WAC, as was previously reported^31^. Interestingly, Eg5, a cellular motor protein, was also co-purified with RNF20, suggesting a physical association of Eg5 with the RNF20/40 complex in cells. The detailed mass spectrometric analysis results are provided in Supplementary Data 1.

To validate affinity purification results, total proteins from HeLa cells were extracted and subjected to co-immunoprecipitation using antibodies against endogenous proteins. Immunoprecipitation with antibodies against RNF20 or RNF40 followed by immunoblotting with antibodies against Eg5 demonstrated that Eg5 was efficiently co-immunoprecipitated with RNF20 and RNF40 (Fig. 1b). Reciprocally, immunoprecipitation with antibodies against Eg5 followed by immunoblotting with antibodies against either RNF20 or RNF40 showed that both RNF20 and RNF40 were co-immunoprecipitated with Eg5 (Fig. 1b). The interaction between the RNF20/40 complex and Eg5 was also detected in human breast carcinoma MCF-7 cells and human pancreatic carcinoma PANC-1 cells using co-immunoprecipitation assays (Fig. 1b).

To further support the observation that the RNF20/40 complex is associated with Eg5 *in vivo*, protein fractionation experiments were carried out through a high-salt extraction and size exclusion approach by fast protein liquid chromatography using Superose 6 size columns. Experimental results revealed that native Eg5 in MCF-7 cells migrated in chromatographic fractions with apparent molecular masses much greater than that of the monomeric or homotetrameric protein (the functional form of Eg5); Eg5 immunoreactivity was detected in fractions with a peak around 669 kDa (Fig. 1c). The pattern of Eg5 in chromatographic fractions largely overlapped with that of RNF20 and RNF40 (Fig. 1c). Eg5 also migrated in fractions with molecular masses around 1,000 kDa. This may be due to a larger complex formation with other proteins in a different phase during cell cycle.

To further investigate the molecular detail involved in the interaction between Eg5 and the RNF20/40 complex, GST pull-down assays were performed using bacterially expressed GST-Eg5 and *in vitro* transcribed/translated RNF20 or RNF40. These experiments showed that Eg5 interacts directly with both RNF20 and RNF40 (Fig. 1d). GST pull-down assays with GST-fused Eg5 truncation mutants, including GST-Eg5N (N-terminal fragment containing motor domain, 1–517 aa), GST-Eg5M (middle region, 518–789 aa), and GST-Eg5C (C-terminal fragment containing microtubule-binding domain, 790–1057 aa) showed that RNF20 interacted with GST-Eg5C, while RNF40 interacted with both GST-Eg5N and GST-Eg5C (Fig. 1e).

### RNF20/40 is co-localized with Eg5 at spindle poles

**Figure 2:**
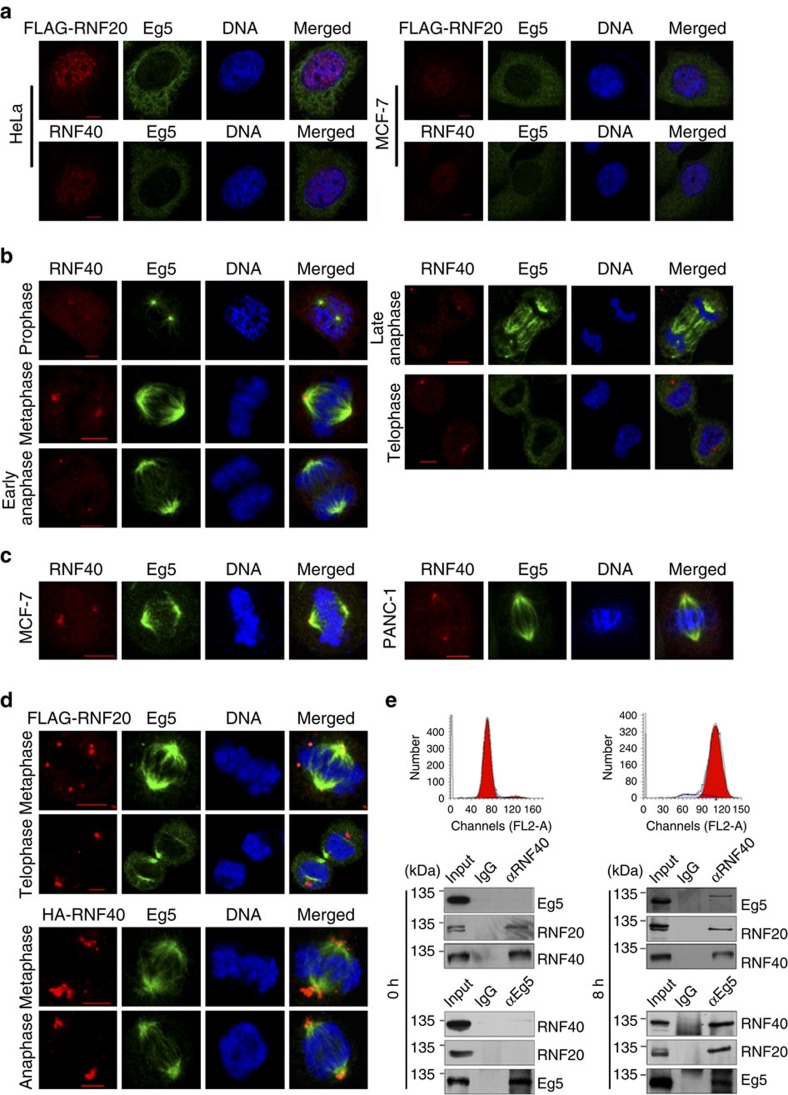
The RNF20/40 complex is co-localized with Eg5 at spindle poles during mitosis. (**a**) Subcellular localization of Eg5, RNF40, and FLAG-RNF20 in interphase HeLa and MCF-7 cells. The distribution of endogenous Eg5 and RNF40 or exogenous FLAG-RNF20 was detected by immunofluorescence assays using anti-RNF40, or anti-FLAG together with anti-Eg5. DAPI staining was conducted to visualize the cell nuclei. Scale bar, 5 μm. (**b**) Co-localization of endogenous RNF40 with Eg5 during different phases of mitosis in HeLa cells. The distribution of endogenous Eg5 and RNF40 was detected using immunofluorescence assays with anti-Eg5 and anti-RNF40. Scale bar, 5 μm. (**c**) Co-localization of endogenous RNF40 with Eg5 at spindle poles during mitosis in MCF-7 and PANC-1 cells. Scale bar, 5 μm. (**d**) Co-localization of overexpressed HA-RNF40 and FLAG-RNF20 with endogenous Eg5 at spindle poles during mitosis. HeLa cells were transfected with HA-RNF40 and FLAG-RNF20 expressing constructs. The localization of Eg5 and overexpressed HA-RNF40 or FLAG-RNF20 was detected by immunofluorescence assays using anti-HA or anti-FLAG together with anti-Eg5. Scale bar, 5 μm. (**e**) Interaction between Eg5 and RNF20/40 during mitosis. HeLa cells were synchronized in G_1_/S phase using double thymidine blocking and collected at 0 and 8 h after release. Immunoprecipitation assays were performed with antibodies against the indicated proteins followed by immunoblotting.

To gain a functional insight into the physical association between the RNF20/40 complex and Eg5, we examined the cellular localization of RNF20/40 and Eg5. Immunofluorescent staining revealed that, in interphase HeLa and MCF-7 cells, the RNF20/40 complex, as the major E3 ligase for H2BK120ub, is predominantly localized in the nucleus, while Eg5, as a microtubule-interacting protein, is mainly found in cytoplasm (Fig. 2a). However, in mitotic HeLa cells, when chromatin is condensed (as indicated by DAPI staining) and the nuclear envelope dissolves, RNF40 was co-localized with Eg5 throughout every stage of mitosis and was especially enriched at spindle poles (Fig. 2b). RNF40 and Eg5 co-localization at mitotic spindle poles was further confirmed by immunofluorescent staining in MCF-7 and PANC-1 cells (Fig. 2c). Since antibodies against RNF20 did not work well for immunofluorescence assays, and to avoid non-specific staining, HeLa cells were transfected with HA-RNF40 or FLAG-RNF20. Immunofluorescent staining with antibodies against hemaglutinin (HA), or FLAG, supports the observation that the RNF20/40 complex is co-localized with Eg5 at spindle poles during mitosis (Fig. 2d).

To verify the co-localization and physical association of the RNF20/40 complex and Eg5 during mitosis, co-immunoprecipitation was performed in interphase cells and mitotic cells. To this end, HeLa cells were synchronized at the G_1_/S phase using double thymidine blocking and then collected at 0 and 8 h after release, when cells were in G_1_/S and G_2_/M phase, respectively (Fig. 2e). The results of immunoprecipitation assays indicated that RNF20/40 was associated with Eg5 in mitotic cells, but not in interphase cells (Fig. 2e). Collectively, these results indicate that the ubiquitin ligase RNF20/40 complex is physically associated with the motor protein Eg5 at spindle poles during mitosis.

### RNF20/40 catalyses the monoubiquitination of Eg5 at K745

**Figure 3:**
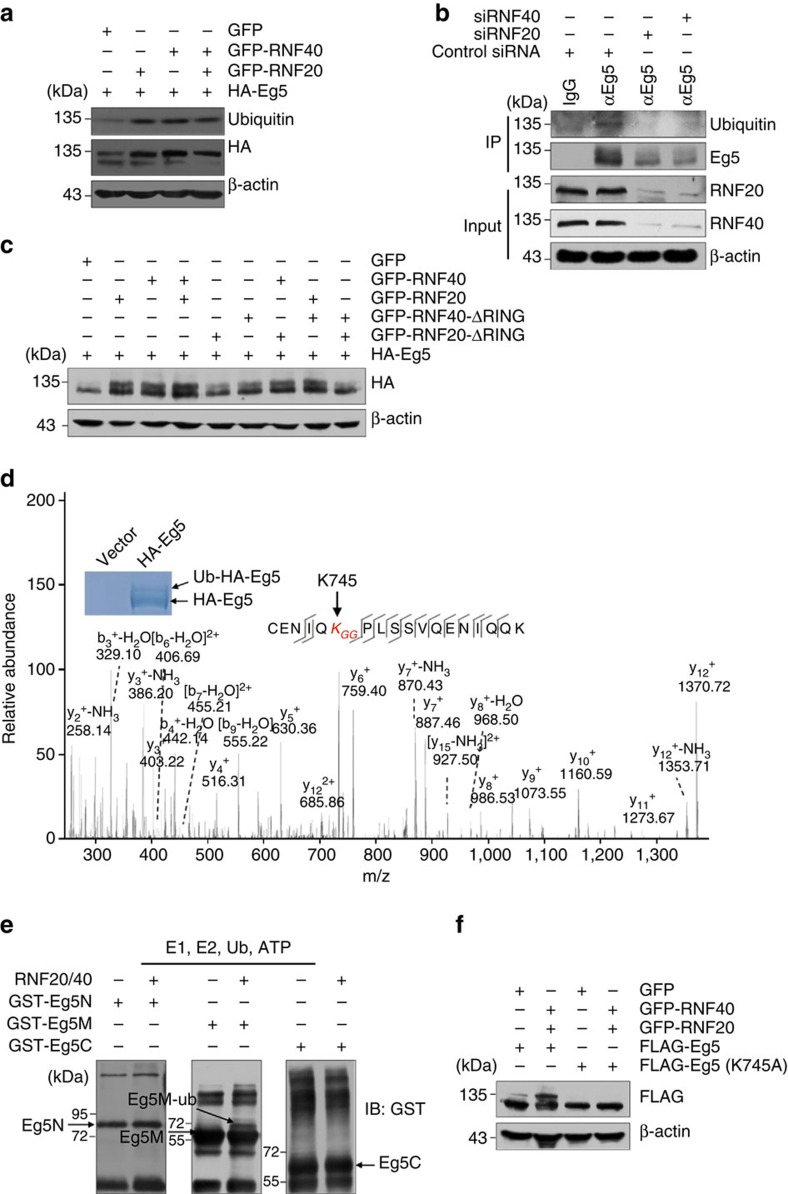
The RNF20/40 complex catalyses the monoubiquitination of Eg5 at lysine 745. (**a**) Overexpression of the RNF20/40 complex increased the monoubiquitination of Eg5. GFP-RNF20 or/and GFP-RNF40 expression constructs were transfected into MCF-7 cells together with HA-Eg5 and Myc-Ubiquitin expression constructs. Immunoprecipitation assays were performed with antibodies against HA, followed by immunoblotting with antibodies against HA and ubiquitin. (**b**) RNF20/40 is essential for the monoubiquitination of endogenous Eg5. Cell lysates from MCF-7 cells transfected with control or RNF20/40 siRNAs were subjected to immunoprecipitation with anti-Eg5 followed by immunoblotting with anti-ubiquitin and anti-Eg5. (**c**) The RING-finger domain of the RNF20/40 complex is essential for catalysing the monoubiquitination of Eg5. MCF-7 cells were co-transfected with the indicated expression constructs and HA-Eg5. Cell lysates were subjected to western blotting with antibodies against the indicated proteins. (**d**) Identification of lysine residue monoubiquitinated in Eg5. Cellular extracts from MCF-7 cells expressing HA-Eg5 were immunopurified with anti-HA affinity columns and eluted with HA peptides. The eluates were resolved by SDS–PAGE and coomassie blue-stained. The protein band indicating Ub-HA-Eg5 on the gel were retrieved and analysed by LCMS/MS. (**e**) Monoubiquitination of Eg5 by the RNF20/40 complex *in vitro*. Recombinant GST-Eg5N, GST-Eg5M or GST-Eg5C was incubated in the presence of E1, E2, Ub and ATP with or without the RNF20/40 complex purified from Sf9 cells, followed by western blotting using antibodies against GST. (**f**) Eg5 is monoubiquitinated at K745 by the RNF20/40 complex. GFP-RNF20 and GFP-RNF40 expression constructs were co-transfected with FLAG-Eg5 or FLAG-Eg5K745A into MCF-7 cells. Cell lysates were subjected to western blotting with antibodies against the indicated proteins.

As stated above, RNF20 and RNF40 contain RING-finger domains and serve as an E3 ligase for H2BK120 monoubiquitination^23^,^24^. The physical association between the RNF20/40 complex and Eg5 prompted us to investigate whether the RNF20/40 complex catalyses the ubiquitination of Eg5. To test this, MCF-7 cells were transfected with HA-Eg5 and Myc-Ubiquitin expression constructs. Cell lysates were then subjected to immunoprecipitation with antibodies against HA followed by immunoblotting with antibodies against ubiquitin and HA. Notably, there was a slowly migrating HA-Eg5 band recognized by both anti-HA, and anti-Ub (Fig. 3a), with a molecular weight approximately equal to a single Ub attached to Eg5, suggesting that Eg5 is monoubiquitinated *in vivo*. When GFP-RNF20 and/or GFP-RNF40 expression constructs were co-transfected with HA-Eg5 and Myc-Ubiquitin expression plasmids into MCF-7 cells, Ub-HA-Eg5 band density increased significantly (Fig. 3a), revealing that RNF20/40 promotes the monoubiquitination of Eg5. In addition, total proteins from MCF-7 cells were extracted and subjected to immunoprecipitation using antibodies against endogenous Eg5 followed by immunoblotting with antibodies against ubiquitin. A band recognized by anti-Ub (Fig. 3b), with a molecular weight approximating a single Ub attached to Eg5 was detected, supporting the notion that endogenous Eg5 is monoubiquitinated *in vivo*. When RNF20 and RNF40 were depleted by their specific small interfering RNAs (siRNAs), Ub-Eg5 density decreased (Fig. 3b), further confirming that RNF20/40 is essential for the monoubiquitination of Eg5.

To further clarify RNF20/40-mediated Eg5 monoubiquitination, truncation mutants of RNF20 (RNF20ΔRING) and RNF40 (RNF40ΔRING) with C-terminal RING-finger domain deleted were generated and these mutants were co-transfected with HA-Eg5 into MCF-7 cells. Western blotting analysis showed that, as long as one component of RNF20/40 is intact and overexpressed in cells, the monoubiquitination of Eg5 increased (Fig. 3c), because both of these two proteins have E3 ligase activity. The results also showed that, compared with wild-type RNF20/40, RNF20ΔRING/RNF40ΔRING failed to enhance the monoubiquitination of Eg5 (the last lane in the up panel, Fig. 3c), suggesting that monoubiquitination of Eg5 depends on the E3 ligase activity of RNF20/40.

To further substantiate RNF20/40-mediated Eg5 monoubiquitination, MCF-7 cells were transfected with HA-Eg5. Cell lysates were then subjected to HA affinity purification and the immunoprecipitates were resolved on SDS–PAGE and coomassie blue stained (Fig. 3d). The protein bands representing Ub-HA-Eg5 and HA-Eg5 on the gel were retrieved and analysed using liquid chromatography-tandem mass spectrometry. We detected a +114-Dalton mass shift for K745 of the Eg5 protein retrieved from the Ub-HA-Eg5 band (Fig. 3d), indicating that Eg5 is monoubiquitinated at K745 in cells. To support RNF20/40-mediated Eg5 monoubiquitination, an *in vitro* ubiquitination assay was performed. To this end, recombinant GST-Eg5N, GST-Eg5M or GST-Eg5C that were purified bacterially were used as substrates, and the RNF20/40 complex which was purified from Sf9 insect cells was supplied as the E3 ligase. The GST-tagged Eg5 truncations were incubated with or without the RNF20/40 complex in an ubiquitination reaction buffer containing E1, E2, Ub and ATP. The reaction was stopped by EDTA and subjected to western blotting using antibodies against GST. The results showed a putative monoubiquitinated Eg5 band when the middle region of Eg5 (containing K745) was used as a substrate, but only in the presence of the RNF20/40 complex (Fig. 3e), further supporting the notion that the RNF20/40 complex catalyses the monoubiquitination of Eg5.

An Eg5 mutant with lysine 745 substituted with alamine (Eg5K745A) was then generated using site-directed mutagenesis. GFP-RNF20 and GFP-RNF40 expression constructs were co-transfected with either FLAG-Eg5 or FLAG-Eg5K745A into MCF-7 cells and western blotting analysis showed that RNF20/40 overexpression significantly enhanced the monoubiquitination of Eg5, but not Eg5K745A (Fig. 3f), supporting the argument that the RNF20/40 complex catalyses the monoubiquitination of Eg5 at K745.

### Monoubiquitination stabilizes Eg5

**Figure 4:**
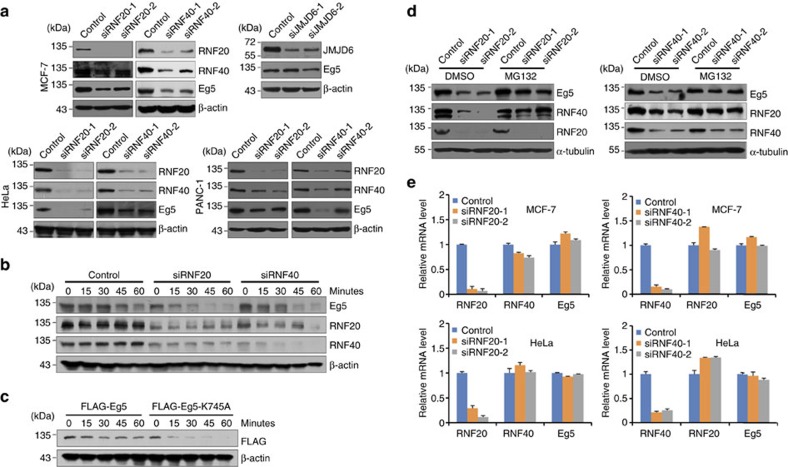
Monoubiquitination of Eg5 at K745 regulates the stability of Eg5. (**a**) Downregulation of Eg5 by RNF20/40 knockdown in MCF-7, HeLa and PANC-1 cells. Total proteins from cells transfected with the control or RNF20/40 siRNAs were extracted and western blotting was performed using antibodies against the indicated proteins. (**b**) The half-life of Eg5 decreased in RNF20/40-depleted cells. HeLa cells were synchronized at G_2_/M phase by nocodazole arrest, and then treated with CHX (50 mg ml^−1^) for 0, 15, 30, 45 and 60 min before they were analysed for the level of Eg5 protein by western blotting using anti-Eg5. (**c**) The half-life of FLAG-Eg5K745A is shorter than that of wild-type Eg5. HeLa cells transfected with FLAG-Eg5 or FLAG-Eg5K745A were synchronized at G_2_/M phase by nocodazole arrest, and then treated with CHX (50 mg ml^−1^) for the designated times before they were analysed for Eg5 expression by western blotting using anti-FLAG. (**d**) The RNF20/40 complex protects Eg5 from proteasome-dependent degradation. Western blotting analysis of the expression of Eg5, RNF20, RNF40 and α-tubulin in MCF-7 cells transfected with indicated siRNAs in the presence or absence of MG132. (**e**) Eg5 is not regulated by the RNF20/40 complex at the transcriptional level. Total mRNA from MCF-7 or HeLa cells transfected with indicated siRNAs was extracted and quantitative real-time RT–PCR assays were performed. Each bar represents the mean±s.d. for triplicate experiments.

Proteins can be monoubiquitinated or polyubiquitinated. Only recently has it become clear that monoubiquitination regulates the location and activity of diverse cellular proteins^32^, and that monoubiquitinated proteins are usually stable^32^. For example, monoubiquitination of histone H2A and H2B regulates transcription and DNA damage response^33^,^34^. Several ion channels and signal-transducing receptors in mammalian cells are monoubiquitinated in response to an extracellular signal, regulating their endocytic transport^35^,^36^. To investigate the biological significance of the RNF20/40 complex-mediated Eg5 monoubiquitination, specific siRNAs against RNF20 or RNF40 were transfected into MCF-7, HeLa and PANC-1 cells. Cell lysates were then subjected to immunoblotting with antibodies against RNF20, RNF40 and Eg5. The results showed both RNF20 and RNF40 knockdown led to a significant reduction of the Eg5 protein level in all three types of cells (Fig. 4a). However, inhibition of JMJD6, a hydroxylase irrelevant to Eg5, made no difference in Eg5 protein level, indicating that Eg5 downregulation by RNF20/40 depletion is specific (Fig. 4a). Consistently, cycloheximide chase assays showed a reduced Eg5 half-life during mitosis in RNF20, or RNF40, -depleted cells (Fig. 4b). Furthermore, the half-life of Eg5K745A is shorter than that of wild-type Eg5 (Fig. 4c), supporting the argument that the monoubiquitination of Eg5 at K745 mediated by the RNF20/40 complex promotes Eg5 stability.

To further investigate the hypothesis that the RNF20/40 complex-mediated Eg5 monoubiquitination protects Eg5 from proteasome-dependent degradation, MCF-7 cells were transfected with RNF20- or RNF40-specific siRNAs and treated with or without MG132. The monoubiquitination of Eg5 decreased in RNF20/40-depleted MCF-7 cells which were treated with MG132 (Supplementary Fig. 1). Western blotting analysis of the expression of Eg5, RNF20, and RNF40 indicated that MG132 could, at least partially, restore Eg5 levels on RNF20 and RNF40 depletion (Fig. 4d). In addition, the downregulation of Eg5 by the RNF20/40 complex knockdown occurred at a post-transcriptional level, as real-time reverse transcription (RT)–PCR analysis showed that neither RNF20 nor RNF40 depletion significantly affected *Eg5* mRNA levels (Fig. 4e). Corroborating this, analysis of a published chromatin immunoprecipitation sequencing data^37^ for H2BK120 monoubiquitination-enriched targets in HeLa cells identified no *Eg5* (Supplementary Data 2). Taken together, these results indicate that the RNF20/40-mediated monoubiquitination promotes Eg5 stability.

In addition, as shown in Fig. 4a, RNF20 knockdown led to a decrease in RNF40 protein level, and RNF40 depletion resulted in decreased RNF20 protein level. And western blotting analysis showed that the downregulation of RNF20 by RNF40 knockdown and the downregulation of RNF40 by RNF20 depletion were both inhibited by MG132 treatment (Fig. 4d). Meanwhile, the experiment of real-time RT–PCR revealed that this reciprocal regulation did not happen at transcriptional level (Fig. 4e). Our results indicated that RNF20 and RNF40 protects each other from proteasome-dependent degradation, and stabilizes their partners by forming a functional complex.

### RNF20/40 participates in spindle assembly through Eg5

**Figure 5:**
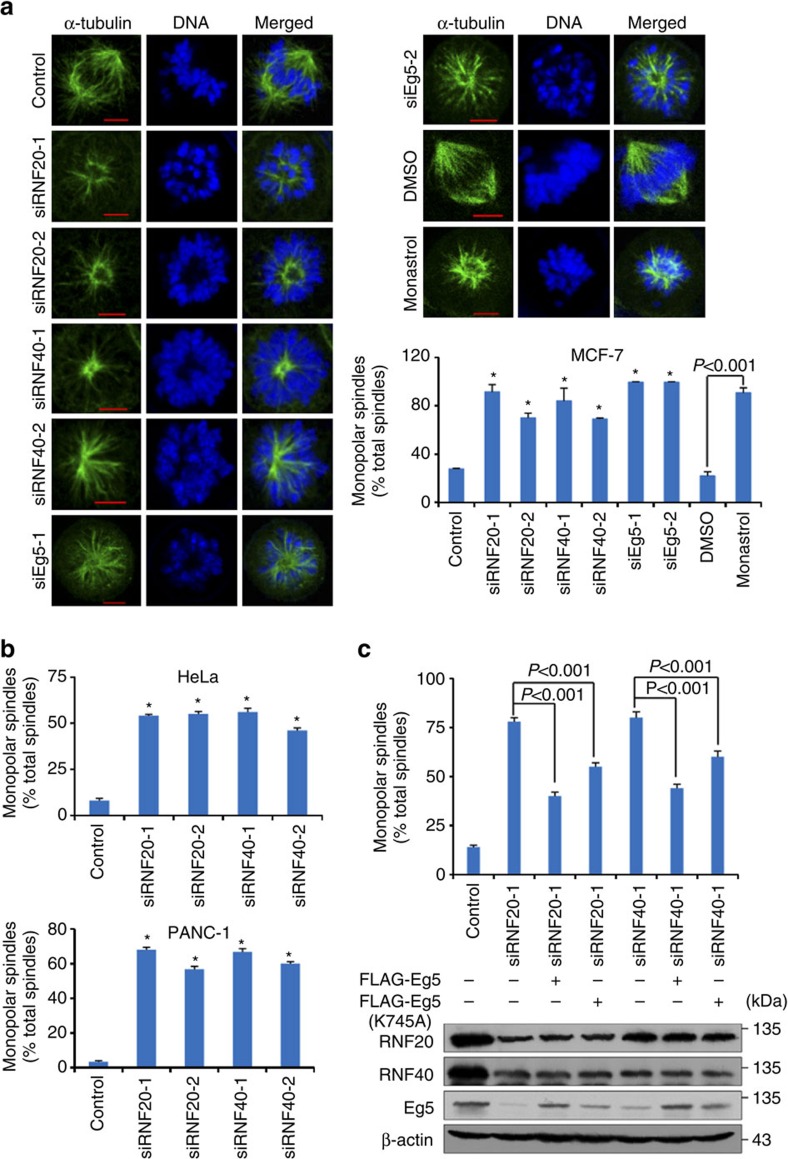
The RNF20/40 complex participates in spindle assembly through stabilizing Eg5. (**a**) Depletion of Eg5, RNF20/40 and inhibition of Eg5 activity leads to monopolar spindle formation. Asynchronous MCF-7 cells treated with indicated siRNAs or 100 μM monastrol were grown on glass coverslips, stained with anti-α-tubulin. DAPI staining was included to visualize the chromosome. Scale bar, 5 μm. According to the morphology of chromosomes, 100 mitotic cells were found out, among which the percentage of monopolar spindles was calculated. Each bar represents the mean±s.d. for triplicate experiments and the *P* value were determined by Student's *t*-test. *P<0.001. Western blotting was performed using antibodies against the indicated proteins. (**b**) Depletion of Eg5, RNF20, or RNF40 leads to monopolar spindle formation in HeLa and PANC-1 cells. The percentage of monopolar spindles was examined by immunofluorescence microscopy. Each bar represents the mean±s.d. for triplicate experiments and the *P* value were determined by Student's *t*-test. *P<0.001. (**c**) Eg5 overexpression efficiently offsets monopolar spindle formation induced by RNF20/40 inhibition. MCF-7 cells with RNF20 or RNF40 depletion were transfected with the empty vector, FLAG-Eg5 or FLAG-Eg5K745A expression constructs, and the percentage of monopolar spindles was examined using immunofluorescence microscopy. Each bar represents the mean±s.d. for triplicate experiments and the *P* value between indicated treatments is examined. Western blotting was performed using antibodies against the indicated proteins.

It is reported that inhibition of Eg5 activity leads to the suppression of centrosome separation in prophase, resulting in the formation of monopolar spindles^12^,^13^. The observations that the RNF20/40 complex is co-localized with Eg5 at spindle poles during mitosis and regulates Eg5 stability prompted us to investigate the role of RNF20/40 in mitotic spindle assembly. To test this, Eg5 was first depleted in MCF-7 cells by its specific siRNAs, or inhibited by its specific inhibitor, monastrol and mitotic spindle assembly was examined using immunofluorescent staining with antibodies against α-tubulin. Consistent with prior observations in HeLa, HCT116, Ptk2, LNCap, RT112 and BS-C1 cells^15^,^17^,^38^, Eg5 depletion and Eg5 activity inhibition was associated with a marked increase in the percentage of MCF-7 cells with a monopolar spindle (Fig. 5a).

RNF20 and RNF40 expression was then knocked down by their specific siRNAs in MCF-7 cells. Immunofluorescent staining with antibodies against α-tubulin revealed that RNF20 or RNF40 depletion led to a significant increase in the percentage of MCF-7 cells with a monopolar spindle (Fig. 5a). Similarly, RNF20 or RNF40 knockdown resulted in a marked increase in monopolar spindle HeLa and PANC-1 cell percentages (Fig. 5b). Consistently, Eg5 overexpression could rescue RNF20 or RNF40 depletion-associated phenotype; the percentage of MCF-7 cells with a monopolar spindle decreased (Fig. 5c). Meanwhile, the rescue efficiency is lower when Eg5K745A was overexpressed due to its lower stability (Fig. 5c). Taken together, these results indicate that the RNF20/40 complex is functionally implemented in mitotic spindle assembly through regulating Eg5 stability.

### RNF20/40-Eg5 is required for cell growth and survival

**Figure 6:**
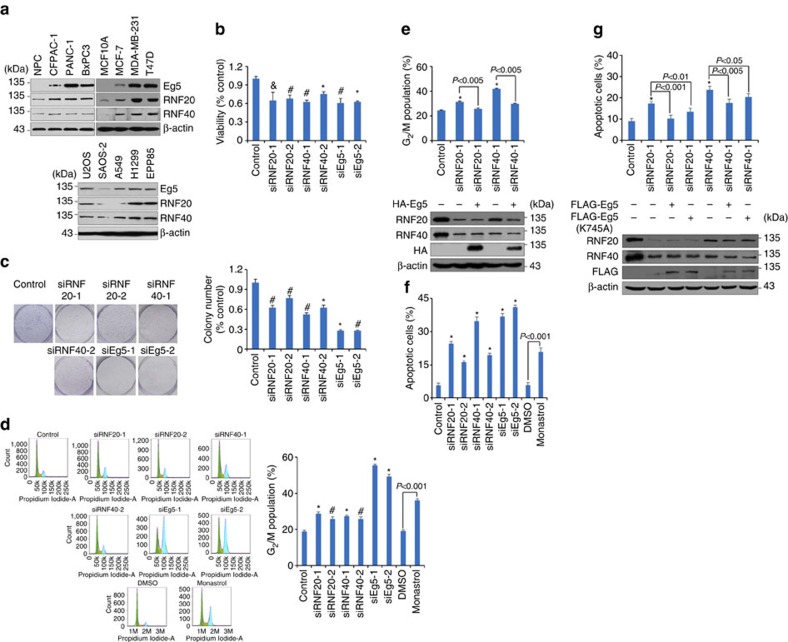
Loss-of-function of RNF20/40 leads to cell G_2_/M arrest and apoptosis via downregulating Eg5. (**a**) The expression of Eg5 and RNF20/40 in different human cells lines. Total proteins from indicated cells were extracted, and western blotting was performed using indicated antibodies. (**b**) Depletion of Eg5 and RNF20/40 decreases the viability of MCF-7 cells. MCF-7 cells were treated with indicated siRNAs for MTT assays. (**c**) Colony formation assay. MCF-7 cells transfected with indicated siRNAs were maintained in culture media for 8 days and stained with crystal violet, and the number of colonies in each condition was counted. (**d**) Depletion of Eg5, RNF20/40 and inhibition of Eg5 activity induced G_2_/M arrest. MCF-7 cells treated with indicated siRNAs or 100 μM monastrol were stained with propidium iodide and subjected to flow cytometry. (**e**) Eg5 overexpression offset RNF20/40 depletion-induced G_2_/M arrest. MCF-7 cells with RNF20 or RNF40 depletion were transfected with the empty vector or HA-Eg5, and the cell cycle was analysed using flow cytometry. The *P* value between the indicated treatments is determined by Student's *t*-test. Western blotting was performed using antibodies against the indicated proteins. (**f**) Depletion of Eg5, RNF20/40 and inhibition of Eg5 led to cell apoptosis. Eg5, RNF20/40-depleted or monastrol-treated MCF-7 cells were stained with FITC-labelled annexin V and propidium iodide, followed by apoptotic analysis using flow cytometry. (**g**) Eg5 overexpression offset RNF20/40 depletion-induced apoptosis. The *P* value between the indicated treatments is determined by Student's *t*-test. Western blotting was performed using antibodies against the indicated proteins. For all the bar charts in Fig. 6, each bar represents the mean±s.d. for triplicate experiments and the *P* value were determined by Student's *t*-test. ^&^*P*<0.01, ^#^*P*<0.005, *P<0.001 versus control.

Mitotic players, such as Bub1, BubR1, Plk1 and Aurora A, are often highly expressed in tumour samples due to an elevated mitotic index in tumour cells^32^,^33^,^34^,^35^,^39^. The importance of the RNF20/40-Eg5 pathway in spindle assembly suggests that RNF20/40 and Eg5 could be implicated in the rapid growth of tumour cells. In fact, the expression of Eg5 is upregulated in various malignancies including lung, bladder and pancreatic cancers^18^,^19^,^20^,^21^. To more fully understand the biological significance of the physical interaction and functional connection between the RNF20/40 complex and the motor protein Eg5 and to explore the potential role of the RNF20/40-Eg5 pathway in tumorigenesis, the expression of RNF20, RNF40 and Eg5 in various human cell lines were profiled. Western blotting analysis of the expression of RNF20, RNF40 and Eg5 in NPC (normal pancreatic cells), CFPAC-1, PANC-1, BxPC3, MCF-10A, MDA-MB-231, MCF-7, U2OS, SAOS-2, A549, H1299 and EPP85 cells showed a positively correlated pattern of expression between the RNF20/40 complex and Eg5 (Fig. 6a). All three protein levels were higher in human breast cancer cells than in the normal human breast epithelial cell line MCF-10A (Fig. 6a), and the expression of Eg5 has been reported to be upregulated by oestrogen in MCF-7 cells^36^, suggesting a role of the RNF20/40 complex and Eg5 in breast carcinogenesis.

The role of RNF20/40 and Eg5 in the growth and proliferation of breast cancer cells was then investigated. Eg5, RNF20 and RNF40 were individually depleted in MCF-7 cells by their specific siRNAs. (3-(4,5)-dimethylthiahiazo (-z-y1)-3,5-di- phenytetrazoliumromide) (MTT) assays and colony formation assays showed that knockdown of either RNF20/40 or Eg5 resulted in a significant decrease in the viability of MCF-7 cells (Fig. 6b,c). In addition, flow cytometry analysis indicated that either RNF20/40 or Eg5 depletion, or Eg5 activity inhibition by monastrol led to a remarkable increase in G_2_/M cell percentages (Fig. 6d). RNF20/40 depletion-induced G_2_/M arrest could be rescued by Eg5 overexpression (Fig. 6e). Moreover, apoptotic analysis, using FITC-labelled annexin V and propidium iodide, showed that either RNF20/40 or Eg5 knockdown, or Eg5 activity inhibition by monastrol promoted apoptosis of MCF-7 cells (Fig. 6f). FLAG-Eg5 overexpression could offset RNF20/40 knockdown-mediated apoptosis, with a higher efficiency than FLAG-Eg5K745A (Fig. 6g). These data indicate that the RNF20/40-Eg5 pathway is essential for the growth and survival of breast cancer cells.

### RNF20/40-Eg5 axis promotes breast carcinogenesis

**Figure 7:**
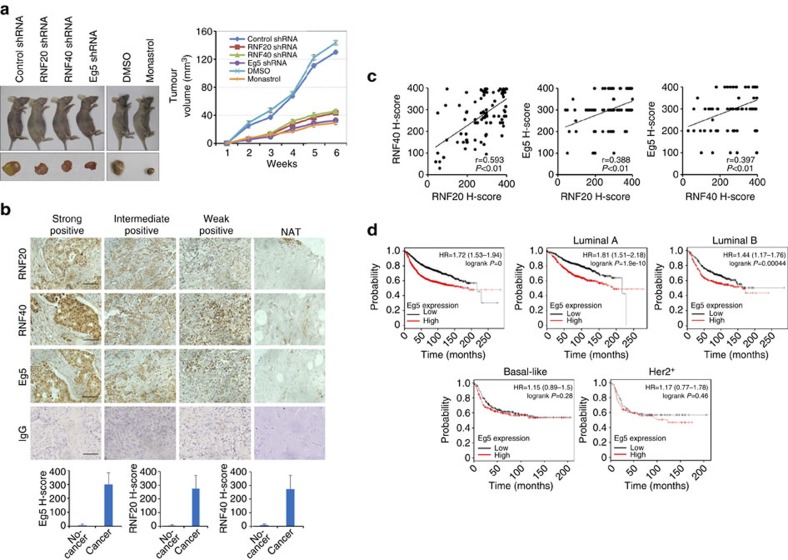
RNF20/40-Eg5 axis in breast carcinogenesis. (**a**) Depletion of Eg5, RNF20/40 and Eg5 inhibition suppressed breast carcinogenesis. MCF-7 cells infected by lentiviruses carrying empty vectors or MCF-7 cells with lentivirus-delivered Eg5, RNF20 or RNF40 knockdown, were transplanted into female athymic nude mice (*n*=6). Another two groups of mice with tumour xenografts developed from normal MCF-7 cells were mock treated or treated with monastrol via intraperitoneal injection. Tumours were measured weekly using a vernier calliper and the volume was calculated according to the formula: V=π/6 × length × width^2^. Each bar represents the mean±s.d. for six animal measurements. (**b**) Immunohistochemical staining of Eg5, RNF20 and RNF40 in breast cancer tissue microarrays. Normal IgG was used as a negative control. Scale bar, 100 μm. The expression of Eg5, RNF20 and RNF40 (*H* score) in 99 breast carcinoma tissues and 10 normal breast tissues was statistically analysed. (**c**) Correlation plot of the expression of Eg5, RNF20 and RNF40 in 99 breast carcinoma tissues. Pearson's correlation analysis was performed, and *P*<0.01 was defined as statistically significant. (**d**) Kaplan–Meier overall survival analysis of Eg5 mRNA expression in 3,458 breast cancer patients from a public data set (http://kmplot.com/analysis/index.php?p=service&cancer=breast).

To further explore the role of the RNF20/40-Eg5 axis in breast carcinogenesis, breast tumours developed from MCF-7 cells were transplanted into nude mice (BALB/c, Charles River, Beijing, China, *n*=6 for each group). The transplanted tumours had either no change in these three genes (infected with lentiviruses carrying an empty vector) or RNF20 knockdown (infected with lentiviruses carrying RNF20 short hairpin RNAs (shRNAs)), RNF40 knockdown (infected with lentiviruses carrying RNF40 shRNAs), or Eg5 knockdown (infected with lentiviruses carrying Eg5 shRNAs). Another two groups of mice with tumour xenografts developed from normal MCF-7 cells were mock treated or treated with monastrol, an Eg5 inhibitor, via intraperitoneal injection. The growth of the implanted tumours was measured over a period of 6 weeks. The results indicated that tumour growth was significantly suppressed in athymic mice which had received tumours with either RNF20, RNF40, Eg5 knockdown or monastrol treatment (Fig. 7a).

To extend these observations into a clinicopathologically relevant setting, immunohistochemistry staining of RNF20, RNF40, and Eg5 proteins in human breast carcinoma samples and adjacent normal tissues was performed. The staining of Eg5 (median *H* score, 299), RNF20 (median *H* score, 274), and RNF40 (median *H* score, 272) was much stronger in 99 breast carcinoma tissues than the staining of these proteins in 10 adjacent normal breast tissues (median *H* score,<20) (Fig. 7b). This suggests a concurrent upregulation of RNF20, RNF40, and Eg5 in breast cancer. Corroborating this notion, the expression of RNF20/40 and Eg5 in these breast carcinoma samples was positively correlated (Fig. 7c). Significantly, Kaplan–Meier survival analysis, using a microarray data set from 3,458 breast cancer patients, revealed high Eg5 mRNA level was significantly associated with worse overall survival (*P*=0) (Fig. 7d), especially for patients with luminal A and B subtype breast tumours. Similar data for RNF20/40 was not available.

## Discussion

When cells enter mitosis, transcription is silenced and transcription-associated proteins are excluded from chromatin. Consequently, the function of transcriptional regulators in mitosis is rarely reported. This study found that, during mitosis, when it is supposed to be excluded from chromatin, the RNF20/40 complex, the major ubiquitin E3 ligase responsible for H2BK120 monoubiquitination during interphase, is physically associated with the motor protein Eg5 and is functionally involved in spindle assembly. This suggests that the RNF20/40 complex is actively involved in mitosis.

Eg5 plays essential roles in spindle assembly in all eukaryotic cells, therefore, an appropriate protein level of Eg5 is important during mitosis. Eg5 inhibition blocks spindle pole body separation and prevents successful mitosis completion^7^,^40^, whereas Eg5 overexpression results in aneuploidy and genetic instability^20^,^41^. However, the regulation of Eg5 stability and activity in mitosis is poorly understood. It was reported that Thr926 phosphorylation in the C-terminal tail catalysed by CDK1 controls Eg5 binding to spindle apparatus^42^. We found in this study that the RNF20/40 complex regulates Eg5 stability by monoubiquitinating it at K745 and protecting it from degradation via the proteasome pathway in mitosis, providing a mechanistic insight into the role of the RNF20/40 complex in spindle assembly.

Mitotic players are often highly expressed in tumour samples due to an elevated mitotic index in tumour cells^32^,^33^,^34^,^35^,^39^. In fact, the expression of Eg5 is upregulated in various malignancies^18^,^19^,^20^,^21^. Eg5 overexpression has been reported to lead to chromosomal missegregation and genomic instability in Eg5 transgenic mice^41^. Chemical compounds inhibiting the activity of Eg5 have emerged as a new generation of anticancer agents that are currently under vigorous clinical investigation^22^,^43^. But the role of this motor protein in breast carcinogenesis has not been reported before. In this study, we found that the expression of Eg5 and RNF20/40 are all elevated in breast carcinomas. Tumour growth in athymic mice receiving tumours with RNF20, RNF40, Eg5 knockdown or Eg5 inhibition, was significantly suppressed, arguing for a role of the RNF20/40-Eg5 axis in breast carcinogenesis. Furthermore, a high Eg5 mRNA level was found to significantly associate with worse overall survival, especially for patients with luminal A and B breast cancers. These results suggest that Eg5 should be explored as prognostic factor for breast cancer, and also be pursued as a potential target for breast cancer treatment. Moreover, our experiments also support pursuing the E3 ligase RNF20/40 as potential target for therapeutic intervention of breast cancer.

## Methods

### Cells and reagents

MCF-7, HeLa and PANC1 cells were all from ATCC (Manassas, VA, USA). Cells were cultured in Dulbecco's modified Eagle's Medium supplemented with 10% fetal bovine serum (HyClone, Logan, Utah, USA), at 37 °C in a humidified atmosphere with 5% CO_2_. Cell lines were authenticated by examination of morphology and growth characteristics and confirmed to be mycoplasma free. The antibodies used against the following proteins were: Eg5 antibodies (sc-374212 for immunofluorescence (1:100), immunoprecipitation and western blotting (1:1,000), sc-66872 for immunohistochemistry (1:50) and western blotting (1:1,000)) from Santa Cruz Biotechnology (Santa Cruz, CA, USA); RNF20 (ab32629, 1:1,000 for WB), RNF40 (ab126959, 1:1,000 for WB) from Abcam, Inc. (Cambridge, MA, USA); HA (4976, 1:1,000 for WB) from Cell Signaling Technology (Danvers, MA, USA); FLAG (M2, F3165, 1:5,000 for WB), ubiquitin (U7258, 1:1,000 for WB) and β-actin (A1978, 1:5,000 for WB) from Sigma-Aldrich (St Louis, MO, USA). Fluorescein- (111-095-003 and 115-095-003, 1:2,000 for IF) or rhodamine-conjugated (111-025-003 and 115-025-003, 1:2,000 for IF) secondary antibodies were obtained from Jackson ImmunoResearch Laboratories (West Grove, PA, USA) and horseradish peroxidase-conjugated secondary antibodies (sc-2030 and sc-2031, 1:5,000 for WB) were from Santa Cruz Biotechnology respectively. Uncropped blots were shown in Supplementary Figs 2–11.

### RNA interference

For siRNA-mediated silencing, RNF20 siRNA-1 (5′-GAUGCAAAUUUCAAGCUCA-3′), RNF20 siRNA-2 (5′-GACAGAUCUUCUUCAGGAA-3′), RNF40 siRNA-1 (5′-CAGCUUAACUCUGGCUACU-3′), RNF40 siRNA-2 (5′-CAGAUGAUGCCACACUCCU-3′), Eg5 siRNA-1 (5′-CAGAUUGAUGUUUACCGAA-3′) and Eg5 siRNA-2 (5′-CUGGAUAUCCCAACAGGUA-3′) was transfected into cells using RNAimax (Invitrogen, Carlsbad, CA, USA) according to the manufacturer's instruction. Scrambled siRNA (5′-UUCUCCGAACGUGUCACGU-3′) was used as a control. All siRNAs were synthesized by Sigma-Aldrich (St Louis, MO, USA).

### Immunohistochemistry

Breast carcinoma and adjacent normal tissue microarray (BC081120) was obtained from US Biomax, Inc. (Rockville, MD, USA). Paraffin-embedded tissue microarrays were deparaffinized in xylene, and then rehydrated with graded alcohols. Antigen retrieval was carried out in 5 mM citrate buffer (pH 6.0) at a sub-boiling temperature for 10 min. After inactivation of endogenous peroxidase in 3% H_2_O_2_ solution in methanol at room temperature for 10 min, the tissue microarrays were blocked with goat serum and incubated with normal IgG, anti-Eg5, anti-RNF20 or anti-RNF40 antibodies. The tissue microarrays were then incubated with biotin-conjugated secondary antibody and streptavidin–biotin–peroxidase. Diaminobenzidine was used as a chromogen substrate to visualize the proteins. Finally, the tissue microarrays were counterstained with haematoxylin, and then observed under microscopy. For the quantitative analysis, a Histo score (*H* score) was calculated based on the staining intensity and percentage of stained cells^44^. The intensity score was defined as follows: 0, no appreciable staining in cells; 1, weak staining in cells comparable with stromal cells; 2, intermediate staining; 3, strong staining; 4, stronger staining. The fraction of positive cells was scored as 0–100%. The *H* score was calculated by multiplying the intensity score and the fraction score, producing a total range of 0–400. Tissue sections were examined and scored separately by two independent investigators blinded to the clinicopathologic data.

### Preparation and treatment of samples for MS analysis

The protein bands were subjected to in-gel digestion according to the standard protocol before mass spectrometer analysis. Briefly, the silver staining bands were excised and washed with 10% Acetic acid/50% EtOH for overnight, and further soaked in HPLC water for 20 min. The gel slices were cut into 1 × 1 mm pieces after destained with 100 mM potassium ferricyanide and 30 mM sodium thiosulfate. The gel slabs were washed with water, 25 mM ammonium bicarbonate in ethanol/water and acetonitrile (ACN), sequentially, and then dried using a SpeedVac for 20 min. Each sample was subjected to reduction and alkylation before in-gel digestion. Dithiothreitol was added into the sample (final concentration of 5 mM) and incubated at room temperature for 45 min. Iodoacetamide was added at a final concentration of 10 mM and incubated at room temperature for 30 min in the dark to carbamidomethylate cysteine residues. After rehydrated, the gel particles were incubated with trypsin (Promega, Madison, Wisconsin, USA) solution (10 μg ml^−1^ in 50 mM NH_4_HCO_3_) and incubated at 37 °C for overnight. Then the liquid fraction containing digested peptides was extracted subsequently with 50%ACN/5%TFA and 75%ACN/0.1%TFA. Finally, after concentrated to complete dryness with a SpeedVac, samples were desalted by C18 ZipTip (Millipore Corporation, Billerica, Massachusetts, USA) before mass spectrometer analysis.

### Nano-HPLC-MS/MS analysis

For identification of protein PTMs, each tryptic digestion was reconstituted in 7 μl of HPLC buffer A (0.1% (v/v) formic acid in water), and 5 μl was injected into a Nano-LC system (EASY-nLC 1000, Thermo Fisher Scientific, Waltham, MA). Each sample was separated by a C18 column (50 μm inner-diameter × 15 cm) with a 105 min HPLC-gradient (linear gradient from 2 to 35% HPLC buffer B (0.1% formic acid in acetonitrile) in 90 min, and then to 90% buffer B in 15 min). The HPLC elute was electrosprayed directly into a Q-Exactive mass spectrometer (Thermo Fisher Scientific, Waltham, MA). The mass spectrometric analysis was carried out in a data-dependent mode, and the parameters were set as follows. The voltage at source was 1.8 kV. For full MS, scan range was from 300 to 1,800 with the resolution of 70,000. The 10 most intense peaks with charge state 2 and above were selected for fragmentation by higher-energy collision dissociation with normalized collision energy of 27%. The MS2 spectra were acquired with 17,500 resolution. The exclusion duration for the data-dependant scan was 18 s, the repeat count was 2, and the exclusion window was set at ±1.5 Da.

To identify proteins associated with FLAG-RNF20, LC-MS/MS analysis was performed using a Thermo Finnigan LTQ linear ion trap mass spectrometer in line with a Thermo Finnigan Surveyor MS Pump Plus HPLC system. Tryptic peptides generated above were loaded onto a trap column (300SB-C18, 5 × 0.3 mm, 5 μm particle; Agilent Technologies, Santa Clara CA) which was connected through a zero dead volume union to the self-packed analytical column (C18, 100 μm i.d × 100 mm, 3 μm particle; SunChrom, Germany). The peptides were then eluted over a gradient (0–45% B in 55 min, 45–100% B in 10 min, where B=80% Acetonitrile, 0.1% formic acid) at a flow rate of 500 nl min^−1^ and introduced online into the linear ion trap mass spectrometer (Thermo Fisher Corporation, San Jose, CA) using nano electrospray ionization (ESI). Data-dependent scanning was incorporated to select the 5 most abundant ions (one microscan per spectra; precursor isolation width 1.0 m z^−1^, 35% collision energy, 30 ms ion activation, exclusion duration: 90 s; repeat count: 1) from a full-scan mass spectrum for fragmentation by collision-induced dissociation.

### Database search-based peptide identification

To identify modifications on Eg5, the resulting MS/MS data were searched against UniProt Human database (9 July 2014 downloaded, 87,724 entries) using Proteome Discoverer software (v1.4) with an overall false discovery rate for peptides of <1%. Peptide sequences were searched using trypsin specificity and allowing a maximum of two missed cleavages. Carbamidomethylation on Cys was specified as fixed modification. GlyGly on lysine, oxidation of methionine and acetylation on peptide N-terminal were fixed as variable modifications. Mass tolerances for precursor ions were set at ±10 p.p.m. for precursor ions and ±0.02 Da for MS/MS. The interesting MS/MS spectra were manually verified.

To identify the identity of proteins associated with FLAG-RNF20, MS data were analysed using SEQUEST (v. 28) against NCBI human protein database (14 December 2011 downloaded, 33,256 entries), and results were filtered, sorted and displayed using the Bioworks 3.2. Peptides (individual spectra) with Preliminary Score (Sp) ≥500; Rank of Sp (RSp) ≤5; and peptides with +1, +2 or +3 charge states were accepted if they were fully enzymatic and had a cross correlation (Xcorr) of 1.90, >2.75 and >3.50, respectively. At least two distinct peptides were assigned to each identified protein. The following residue modifications were allowed in the search: carbamidomethylation on cysteine as fix modification and oxidation on methionine as variable modification. Peptide sequences were searched using trypsin specificity and allowing a maximum of two missed cleavages. Sequest was searched with a peptide tolerance of 3 Da and a fragment ion tolerance of 1.0 Da.

### Animal experiments

The targeting sequences of siRNF20-1, siRNF20-2, siRNF40-1, siRNF40-2, siEg5-1 and siEg5-2 were separately cloned into pLL3.7 lentiviral vectors according to the manufacturer's instruction. The recombinant construct, together with three assistant vectors (pRRE, VSVG and RSV/REV), were then transiently transfected into HEK 293T cells. Viral supernatants were collected both 24, and 48, hour later, clarified by filtration, and concentrated by ultracentrifugation. Two kinds of lentiviruses which express efficient shRNAs targeting the same gene (for example, shRNF20-1 and shRNF20-2) were jointly employed to infect MCF-7 cells which were then injected subcutaneously into the right flanks of female athymic nude mice (BALB/c, Charles River; between five and six weeks of age; six mice per group). Two other groups of mice (BALB/c, Charles River; between five and six weeks of age; six female mice per group) with tumour xenografts developed from normal MCF-7 cells were mock treated or treated with 5 mg kg^−1^ monastrol by intraperitoneal injection. Tumour volume was measured once a week using a vernier caliper and calculated according to the following formula: V=π/6 × length × width^2^. Tumour volume was examined by an investigator blinded to the treatment procedure. Mice in which tumours did not form would be removed from the study. Tumours were observed in all the mice injected with MCF-7 cells. The mice were sacrificed 6 weeks post injection. Tumours were then isolated and photographed. The sample replicates were treated as random effects. We did not select a specific time point to assess significance. Animal handling and procedures were approved by Institutional Animal Care and Use Committees of Tianjin Medical University.

### Statistical analysis

*P* values were determined by the two-tailed Student's *t*-test. Error bars in figures represent s.d. for at least triplicate experiments.

### Data availability

The Kaplan–Meier overall survival analysis of Eg5 mRNA expression in breast cancer patients is available on a public data set (http://kmplot.com/analysis/index.php?p=service&cancer=breast). The authors declare that all other data supporting the findings of this study are available within the article and its Supplementary Information Files, or available from the corresponding author on request.

## Additional information

**How to cite this article:** Duan, Y. *et al*. Ubiquitin ligase RNF20/40 facilitates spindle assembly and promotes breast carcinogenesis through stabilizing motor protein Eg5. *Nat. Commun.* 7:12648 doi: 10.1038/ncomms12648 (2016).

## Supplementary Material

Supplementary Information Supplementary Figures 1-11

Supplementary Data 1 Mass Spectrometry Analysis of RNF20-containing Protein Complex. Cellular extracts from HeLa cells stably expressing FLAG-RNF20 were immunopurified with anti-FLAG affinity columns and eluted with FLAG peptides. The eluates were resolved by SDS-PAGE and silver-stained. The detailed information of the mass spectrometric analysis of the protein bands is listed.

Supplementary Data 2 H2B-K120 monoubiquitination-enriched target genes. A published ChIP-seq data (GSM818830) of H2BK120 monoubiquitination in HeLa cells was analyzed to identify target genes whose promoter is occupied by H2BK120ub. The information of these target genes is listed.
