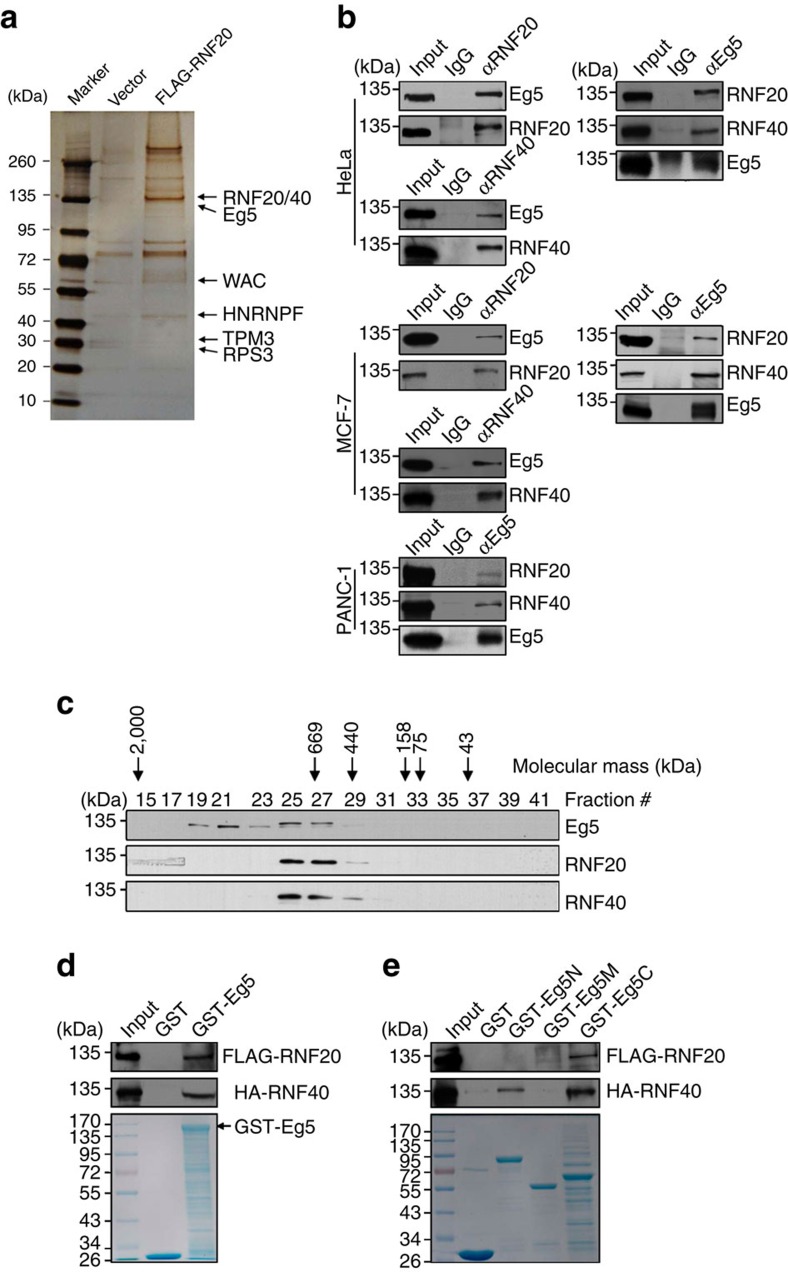# Corrigendum: Ubiquitin ligase RNF20/40 facilitates spindle assembly and promotes breast carcinogenesis through stabilizing motor protein Eg5

**DOI:** 10.1038/ncomms13462

**Published:** 2016-11-04

**Authors:** Yang Duan, Dawei Huo, Jie Gao, Heng Wu, Zheng Ye, Zhe Liu, Kai Zhang, Lin Shan, Xing Zhou, Yue Wang, Dongxue Su, Xiang Ding, Lei Shi, Yan Wang, Yongfeng Shang, Chenghao Xuan

Nature Communications **7**: Article number: 12648; DOI: 10.1038/ncomms12648 (2014); Published: 08 25 2016; Updated: 11 04 2016

In Fig. 1a of this Article, the locations of labels for TPM3 and RPS3 protein bands are incorrect. The correct version of Fig. 1 appears below.

## Figures and Tables

**Figure 1:**