# Supplementary material for: Ubiquitin ligase RNF20/40 facilitates spindle assembly and promotes breast carcinogenesis through stabilizing motor protein Eg5
Source: Nat Commun. 2016 Aug 25;7:12648. doi: 10.1038/ncomms12648 (PMC5007379; doi:10.1038/ncomms12648)
Supplement: Supplementary Information — Supplementary Figures 1-11 [file ncomms12648-s1.pdf]

Supplementary Figure 1

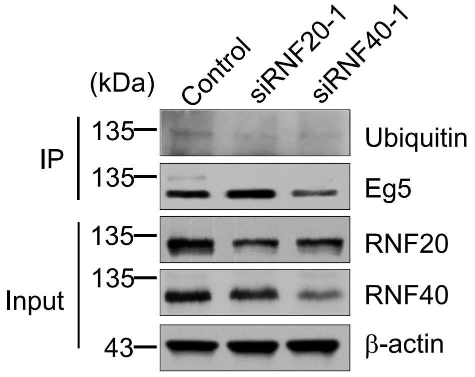

Supplementary Figure 1. The monoubiquitination of Eg5 is decreased upon RNF20/40 depletion. MCF-7 cells transfected with control or RNF20/40 siRNAs were treated with MG132, and then cell lysates were subjected to immunoprecipitation with anti-Eg5 followed by immunoblotting with anti-ubiquitin and anti-Eg5.

Supplementary Figure 2

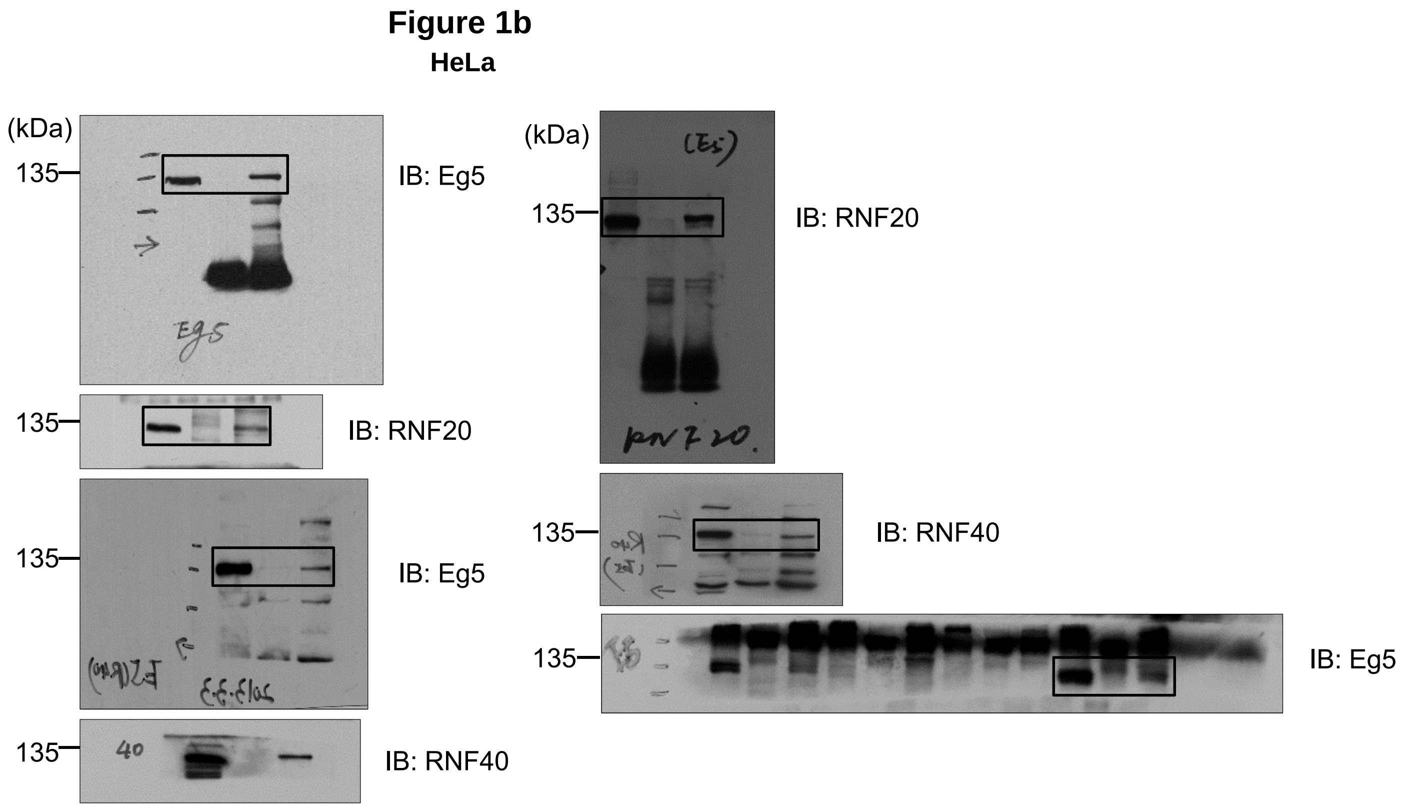

Supplementary Figure 2. Uncropped scans of blots. Uncropped scans of Figure 1b (IPs in HeLa cells).

Supplementary Figure 3

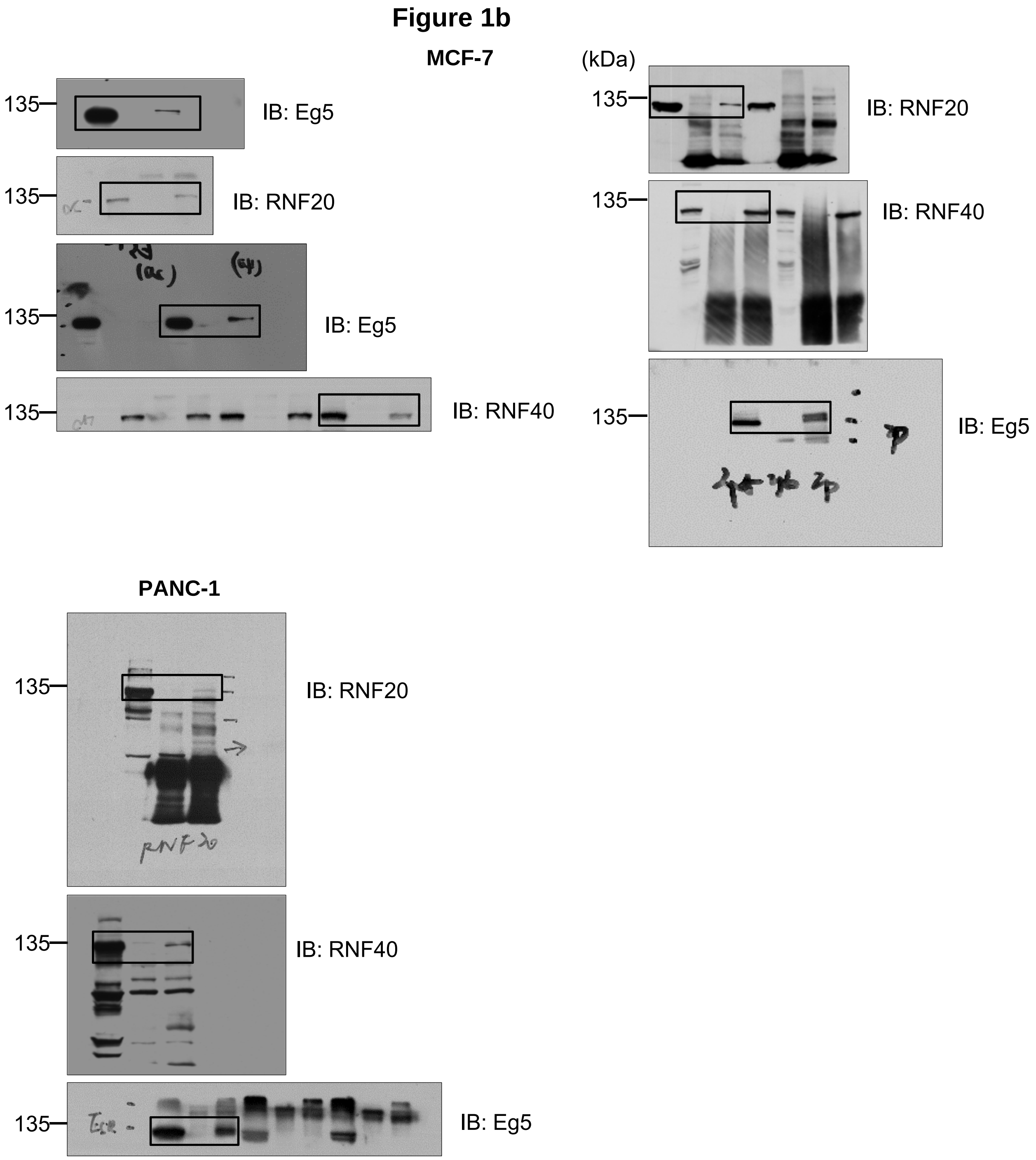

Supplementary Figure 3. Uncropped scans of blots. Uncropped scans of Figure 1b (IPs in MCF-7 and PANC-1 cells).

Supplementary Figure 4

Figure 1c

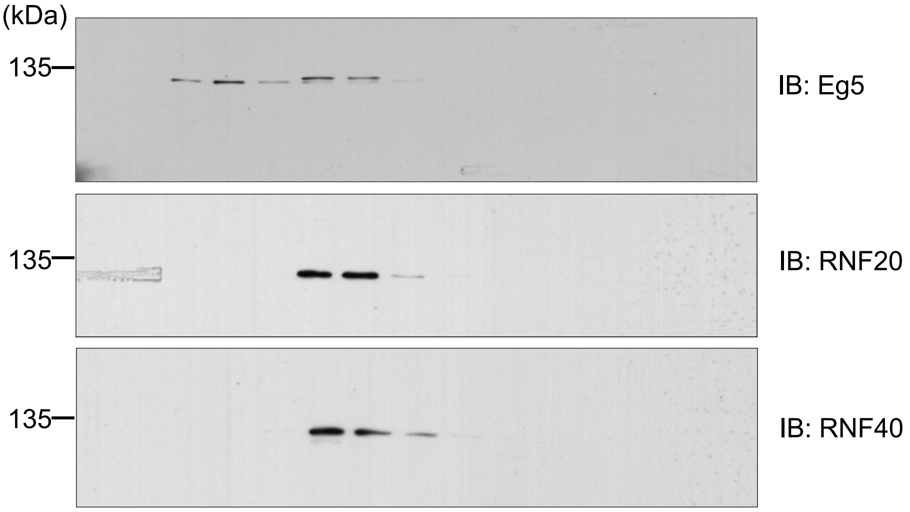

Figure 1d

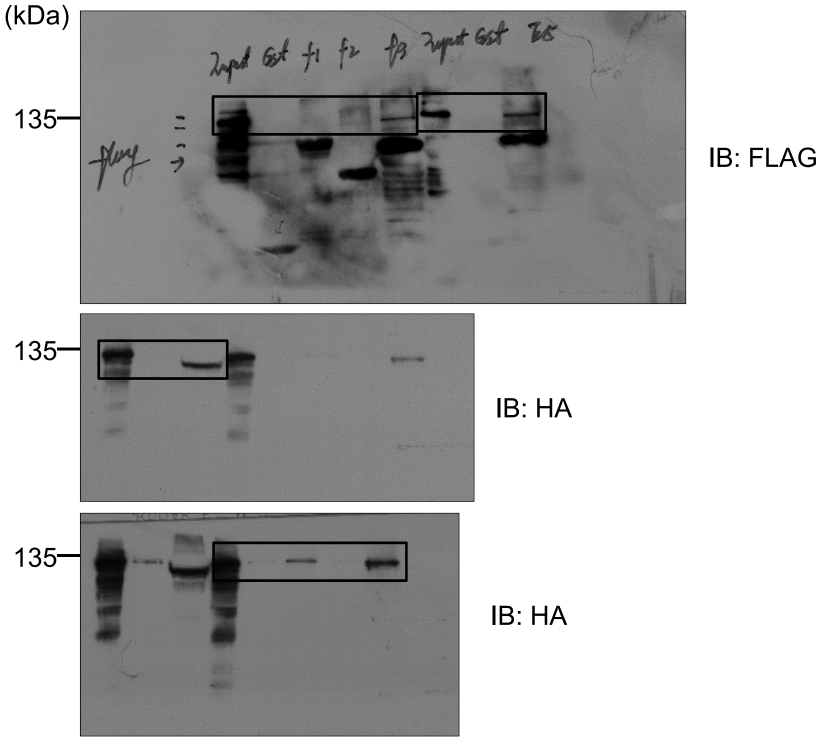

Supplementary Figure 4. Uncropped scans of blots. Uncropped scans of Figure 1c and Figure 1d.

Supplementary Figure 5

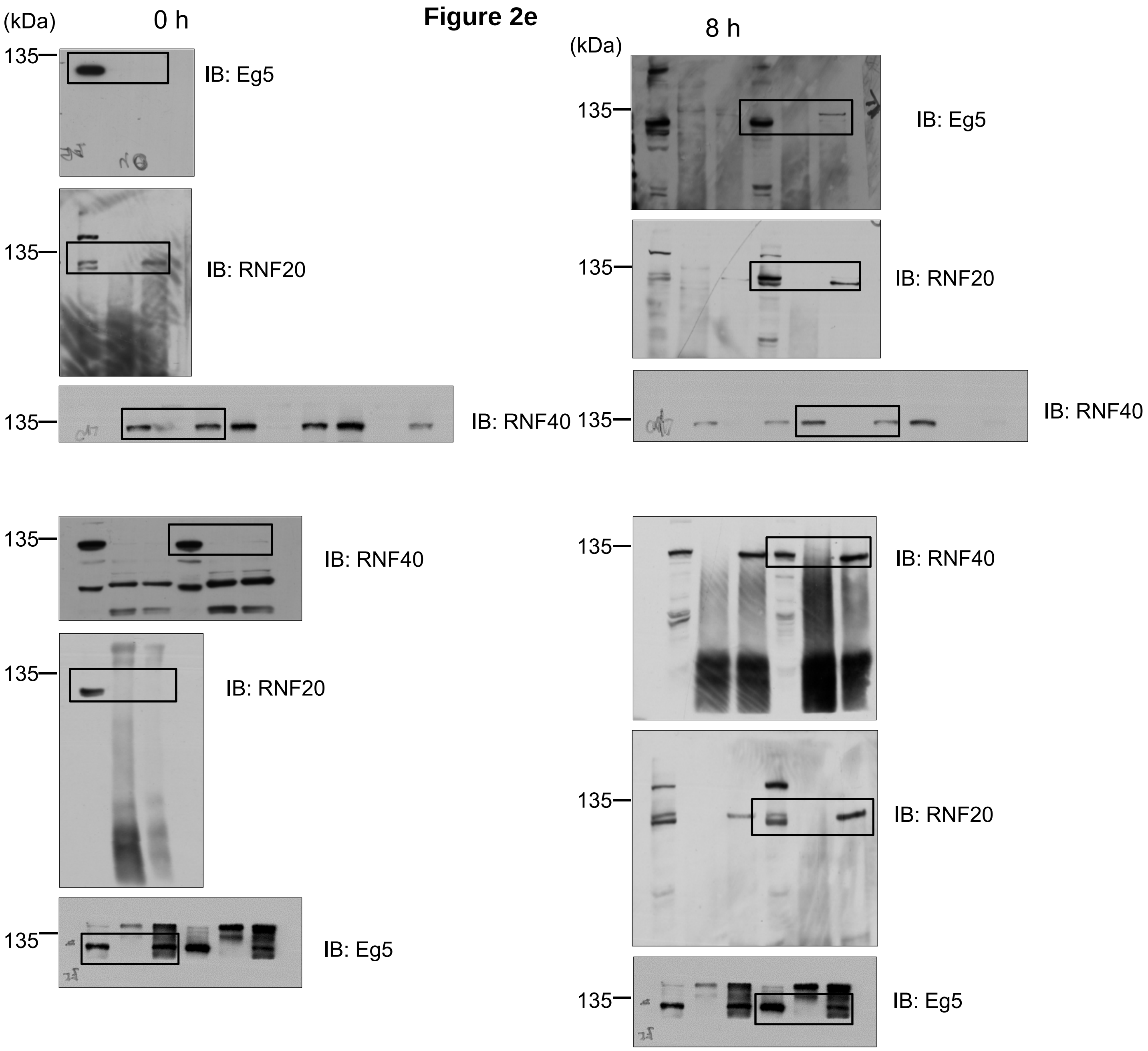

Supplementary Figure 5. Uncropped scans of blots. Uncropped scans of Figure 2e.

Supplementary Figure 6

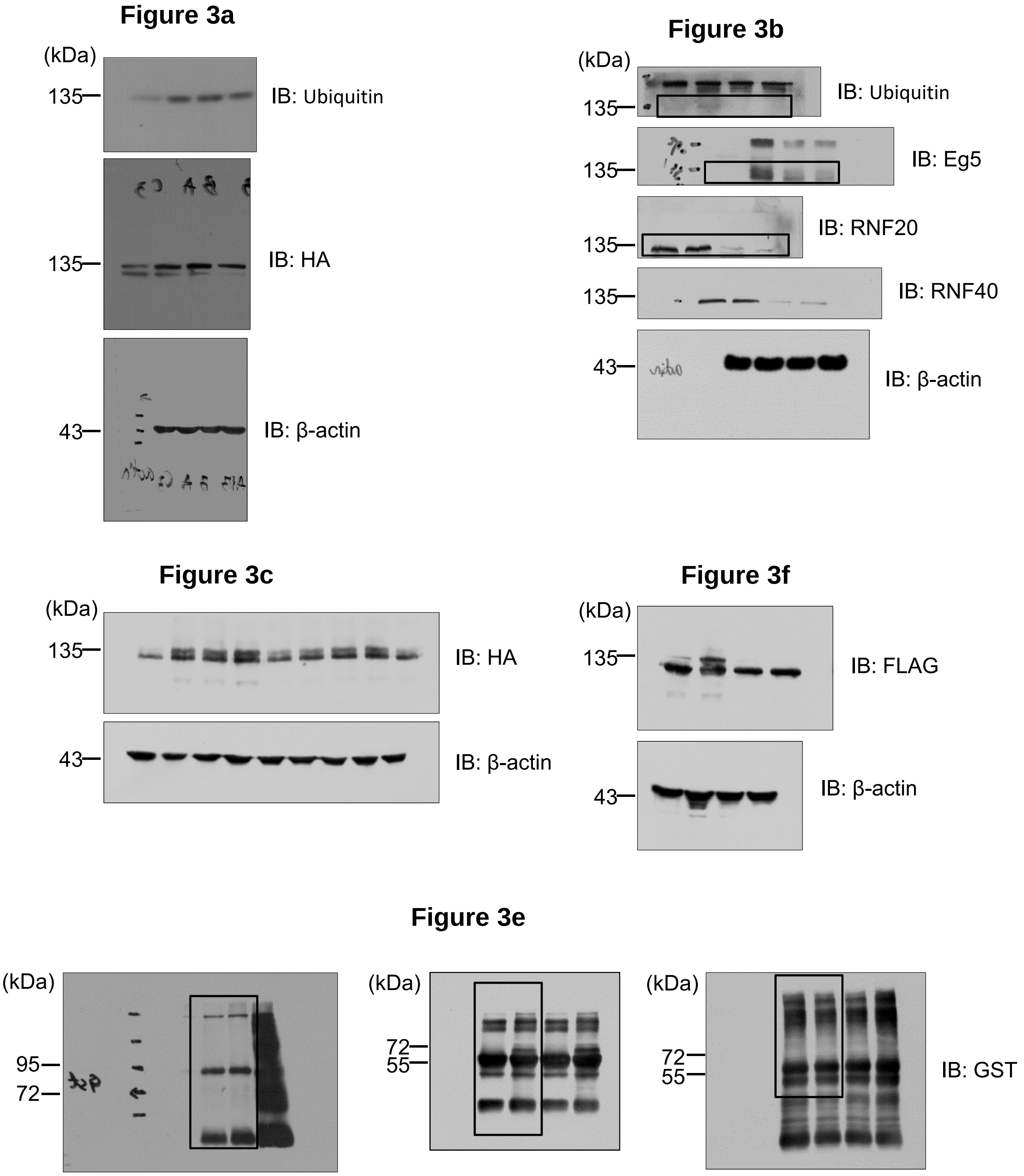

Supplementary Figure 6. Uncropped scans of blots. Uncropped scans of Figure 3a, Figure 3b, Figure 3c, Figure 3e, and Figure 3f.

Supplementary Figure 7

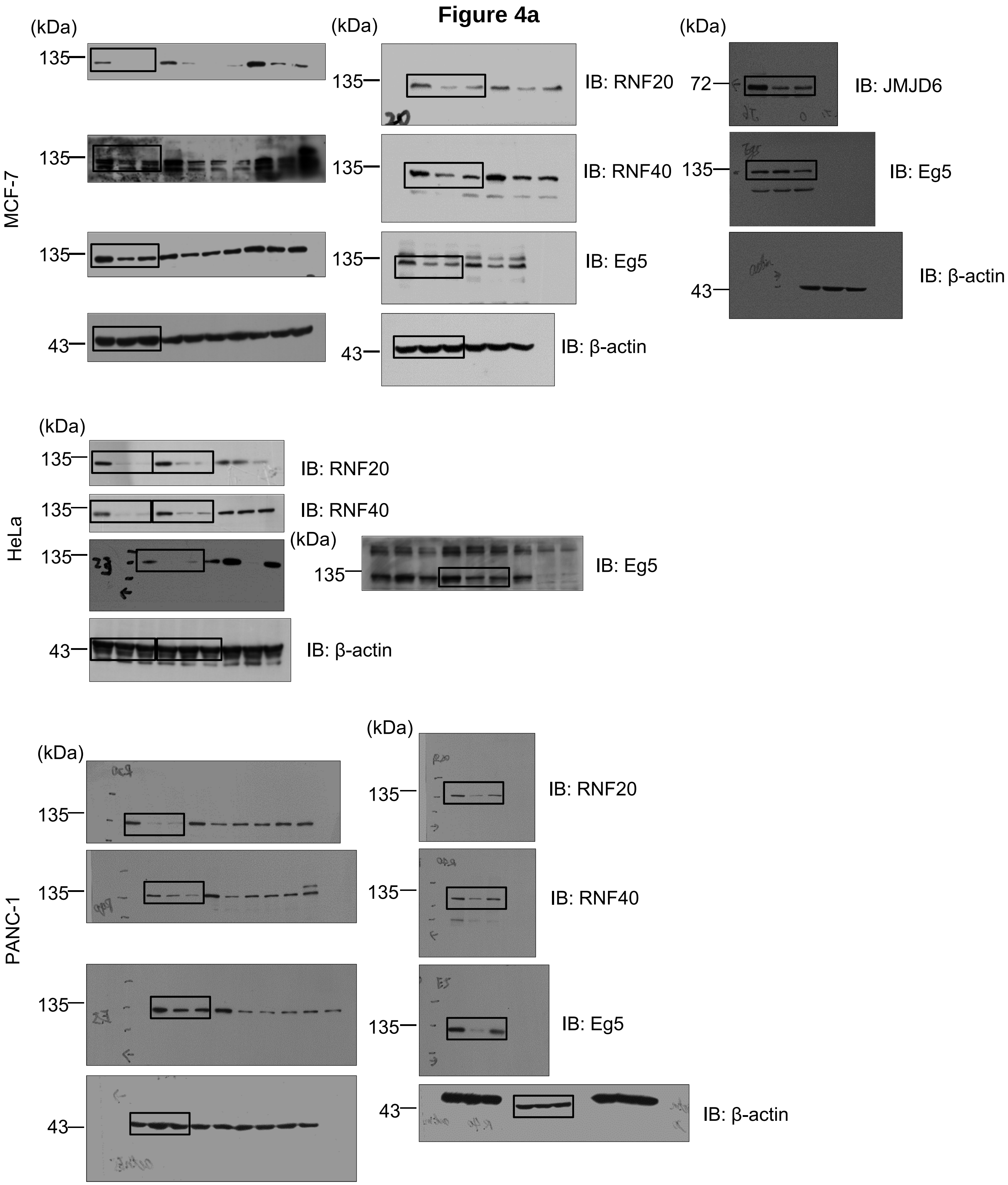

Supplementary Figure 7. Uncropped scans of blots. Uncropped scans of Figure 4a.

Supplementary Figure 8

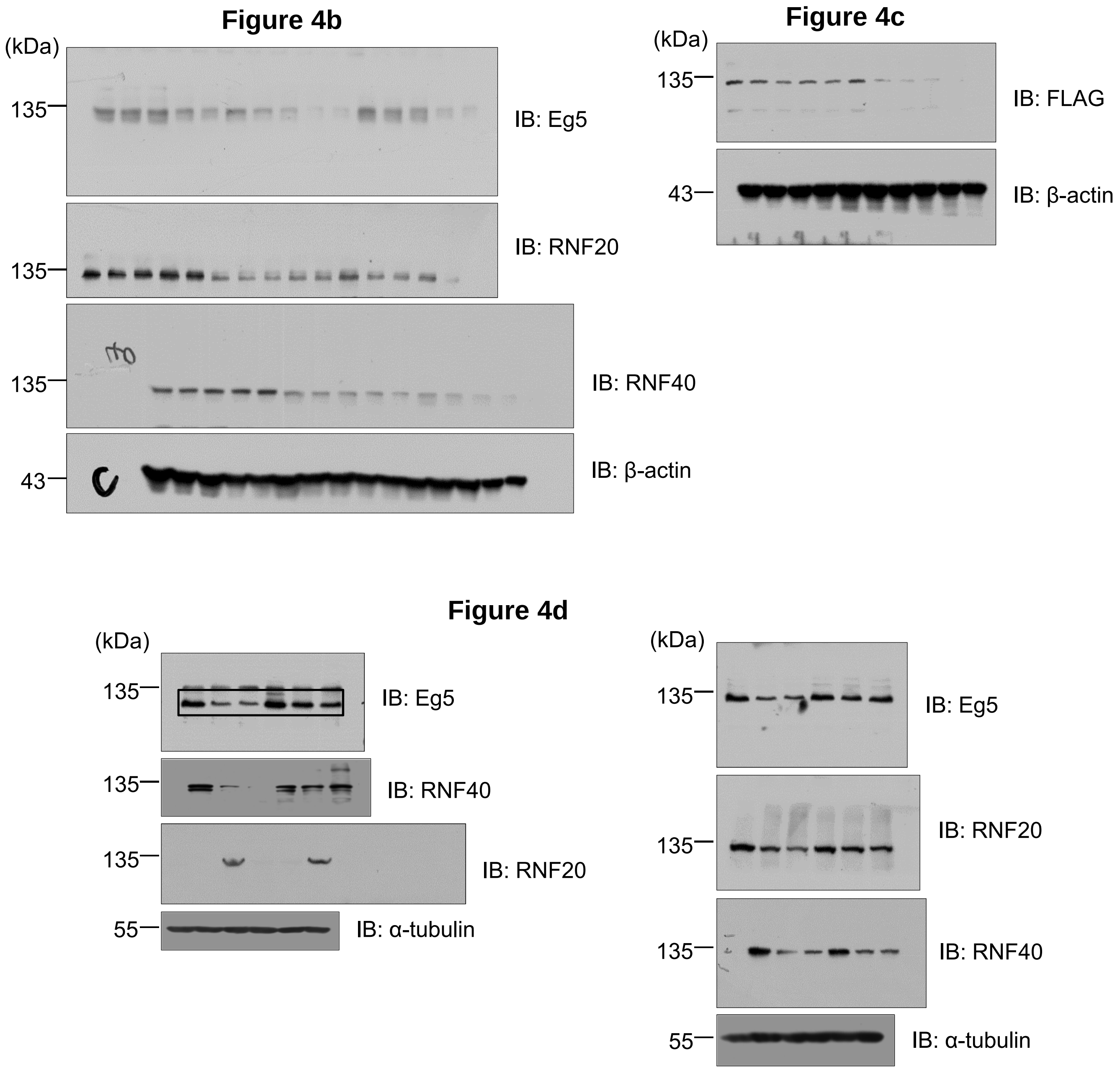

Supplementary Figure 8. Uncropped scans of blots. Uncropped scans of Figure 4b, Figure 4c, and Figure 4d.

Supplementary Figure 9

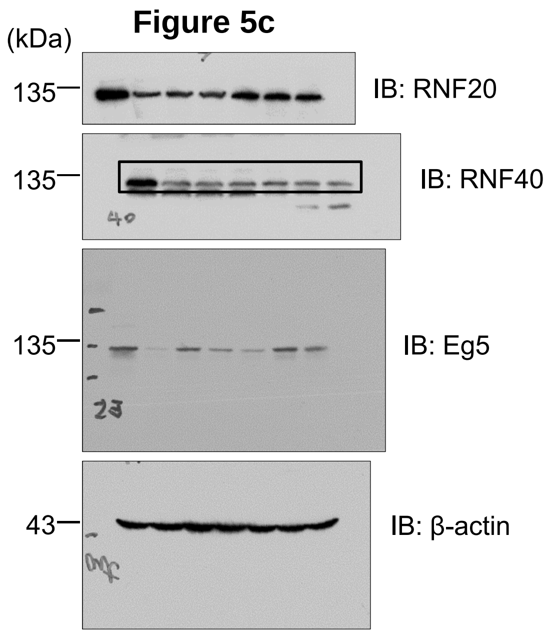

Supplementary Figure 9. Uncropped scans of blots. Uncropped scans of Figure 5c.

Supplementary Figure 10

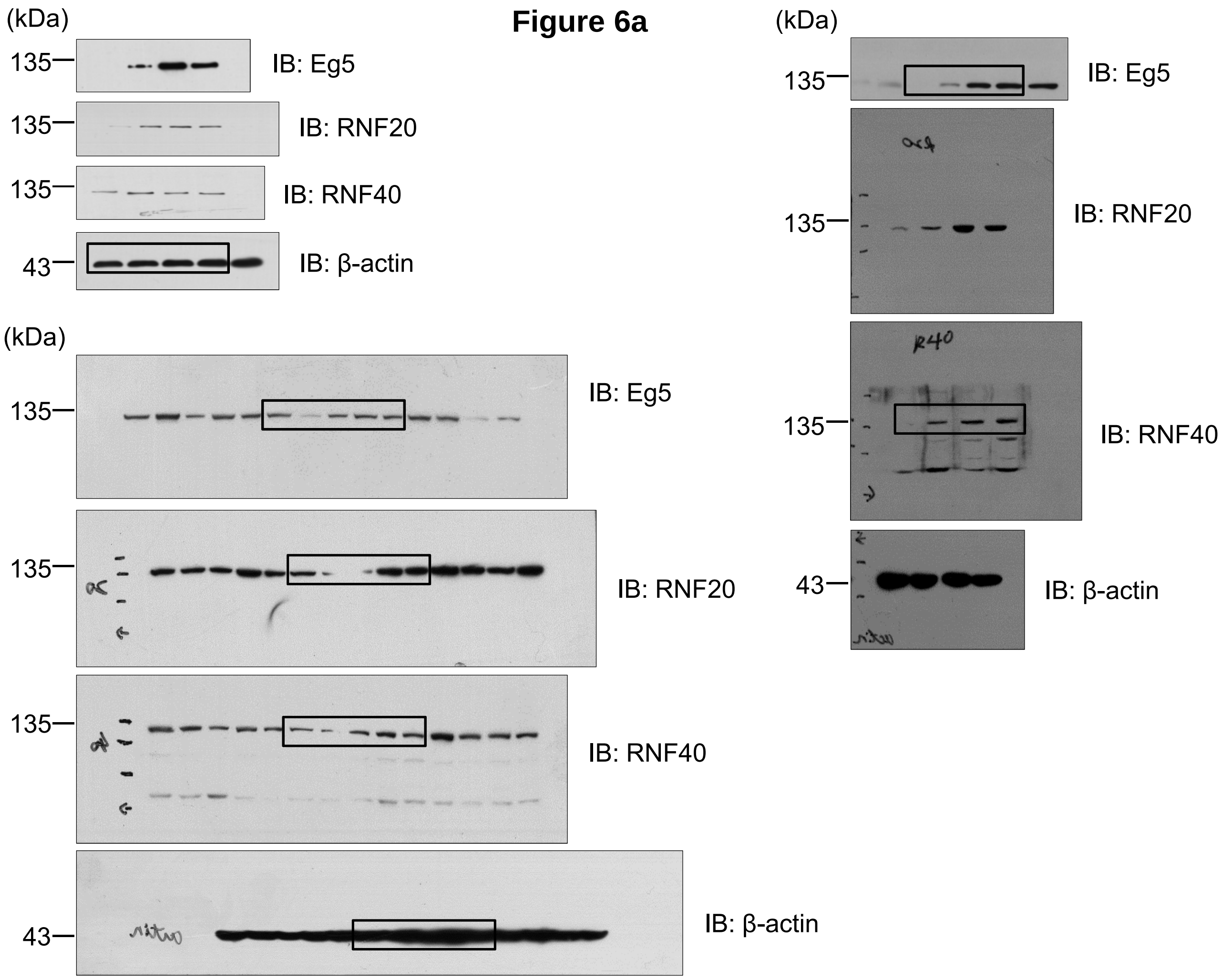

Supplementary Figure 10. Uncropped scans of blots. Uncropped scans of Figure 6a.

Supplementary Figure 11

Figure 6e

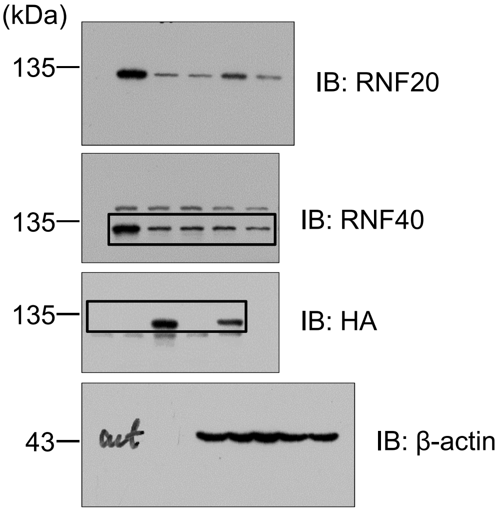

Figure 6f

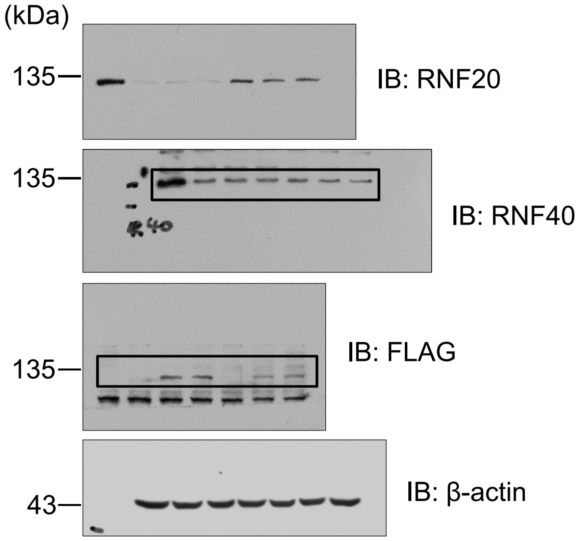

Supplementary Figure 1

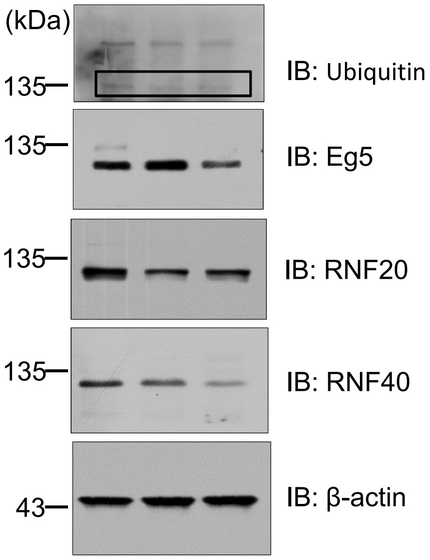

Supplementary Figure 11. Uncropped scans of blots. Uncropped scans of Figure 6e, Figure 6f, and Supplementary Figure 1.
